# Icosapent ethyl–induced lipoprotein remodeling and its impact on cardiovascular disease risk markers in normolipidemic individuals

**DOI:** 10.1172/jci.insight.193637

**Published:** 2025-10-08

**Authors:** Lauri Äikäs, Petri T. Kovanen, Martina B. Lorey, Reijo Laaksonen, Minna Holopainen, Hanna Ruhanen, Reijo Käkelä, Matti Jauhiainen, Martin Hermansson, Katariina Öörni

**Affiliations:** 1 Wihuri Research Institute, Helsinki, Finland.; 2 Molecular and Integrative Biosciences Research Programme, Faculty of Biological and Environmental Sciences, University of Helsinki, Helsinki, Finland.; 3 Zora Biosciences Oy, Espoo, Finland.; 4 Finnish Cardiovascular Research Center – Tampere, Tampere University, Tampere, Finland.; 5 Helsinki University Lipidomics Unit (HiLIPID), Helsinki Institute of Life Science (HiLIFE), Biocenter Finland, University of Helsinki, Helsinki, Finland.; 6 Minerva Foundation Institute for Medical Research, Helsinki, Finland.

**Keywords:** Clinical Research, Metabolism, Vascular biology, Cardiovascular disease, Lipoproteins, Metabolomics

## Abstract

**BACKGROUND:**

Icosapent ethyl (IPE), an ethyl ester of eicosapentaenoic acid (EPA), reduces cardiovascular disease (CVD), but the mechanism remains elusive. We examined the effect of IPE supplementation on lipoprotein subclasses, lipidomes, and pro-atherogenic properties.

**METHODS:**

Using 3 independent metabolomic platforms, we examined the effect of high-dose IPE supplementation for 28 days on fatty acid profiles, lipoprotein subclasses, lipidomes, and pro-atherogenic properties in normolipidemic volunteers (*n* = 38).

**RESULTS:**

IPE supplementation increased lipoprotein EPA on average 4-fold within 7 days, returning to baseline after a 7-day washout. Notably, the incorporation displayed marked interindividual variance, negatively correlating with baseline levels. We identified persistent participant-specific lipoprotein fingerprints despite uniform IPE-induced lipidome remodeling across all lipoprotein classes. This remodeling resulted in reductions in saturated, monounsaturated, and n-6 polyunsaturated fatty acids, resulting in reduced clinical risk markers, including triglyceride, remnant cholesterol, and apolipoprotein B (apoB) levels and 10-year CVD risk score. Of the pro-atherogenic properties tested, IPE significantly reduced apoB lipoprotein binding to proteoglycans, which correlated with lower apoB particle concentration, cholesterol content, and specific lipid species in LDL, including phosphatidylcholine 38:3 previously associated with CVD.

**CONCLUSION:**

These findings highlight IPE’s rapid, uniform remodeling of lipoproteins and reduced proteoglycan binding, likely contributing to previously observed CVD risk reduction. Persistent interindividual lipidome signatures underscore the potential for personalized therapeutic approaches in atherosclerotic CVD treatment.

**TRIAL REGISTRATION:**

NCT04152291.

**FUNDING:**

Jenny and Antti Wihuri Foundation, Research Council of Finland, Sigrid Jusélius Foundation, Finnish Foundation for Cardiovascular Research, Emil Aaltonen Foundation, Ida Montin Foundation, Novo Nordisk Foundation, Finnish Cultural Foundation, and Jane and Aatos Erkko Foundation.

## Abstract 2

Icosapent ethyl, an omega-3 therapy, rapidly remodels lipoproteins and lowers cardiovascular disease risk markers. Individual differences suggest potential for personalized approaches to cardiovascular disease prevention.

## Graphical abstract

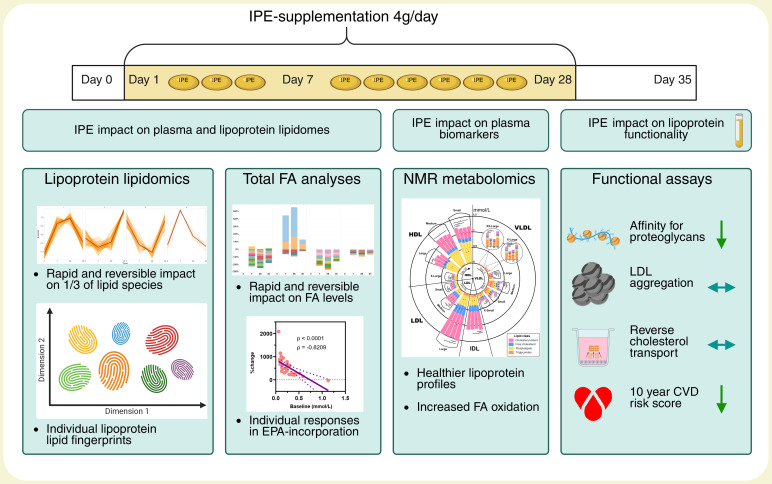

## Introduction

Cardiovascular disease (CVD) remains a leading cause of death worldwide, driving the ongoing search for effective preventative strategies. Among the promising approaches is the use of long-chain omega-3 polyunsaturated fatty acids (n-3 PUFAs), particularly eicosapentaenoic acid (EPA), commonly administered in its ethyl ester form, icosapent ethyl (IPE). Large clinical trials, such as REDUCE-IT and JELIS, have demonstrated that high-dose EPA supplementation significantly reduces CVD events and mortality (1, 2). As a result, EPA has been approved, when used in combination with statins, to reduce cardiovascular risk in hypertriglyceridemic high-risk patients (3). However, the precise mechanisms by which EPA exerts its cardioprotective effects, including its distribution among plasma lipoproteins, remain unclear.

Lipoproteins — complex particles responsible for lipid (including EPA) transport in the bloodstream — are causal to the pathogenesis of atherosclerotic CVD (ASCVD). Key events in the initiation of ASCVD are the retention, modification, and aggregation of apolipoprotein B–containing (apoB-containing) lipoproteins within the arterial intima, driven by their interactions with extracellular matrix proteoglycans (4–7). The structural and compositional characteristics of lipoproteins, particularly LDL, dictate their affinity for proteoglycans (8–10) and their propensity for harmful modifications and aggregation (9, 11–13), processes that contribute to chronic inflammation, plaque instability, and subsequent cardiovascular events (6, 7).

Recent studies suggest substantial variability in lipoprotein characteristics among individuals, with certain types of particles showing a greater propensity to aggregate and bind to proteoglycans, thereby posing a higher risk for ASCVD (5, 11, 13). Furthermore, lipoproteins are not static entities but are dynamically remodeled in response to dietary (14, 15) and pharmacological interventions (16, 17) or disease (17–21). Such remodeling can profoundly alter the lipid composition within lipoprotein particles, potentially affecting their atherogenic potential (9, 12).

Understanding the extent of lipoprotein remodeling by EPA, as well as the distribution of EPA within different lipoprotein fractions, is crucial, as it may influence not only lipid metabolism but also the mechanisms through which EPA exerts its protective effects against ASCVD. While fish oil products, containing EPA, are known to reduce triglyceride (TG) levels by inhibiting hepatic VLDL secretion (22–24) and increasing lipoprotein lipase (LPL) activity (25), these mechanisms alone may not fully explain the observed reduction in CVD events. EPA’s influence on lipoprotein lipid composition, particularly concerning binding, aggregation, and reverse cholesterol transport, remains an area requiring further investigation. Insights into these mechanisms could enhance our understanding of EPA’s cardioprotective effects and contribute to more personalized approaches in CVD prevention.

## Results

**Figure 1:**
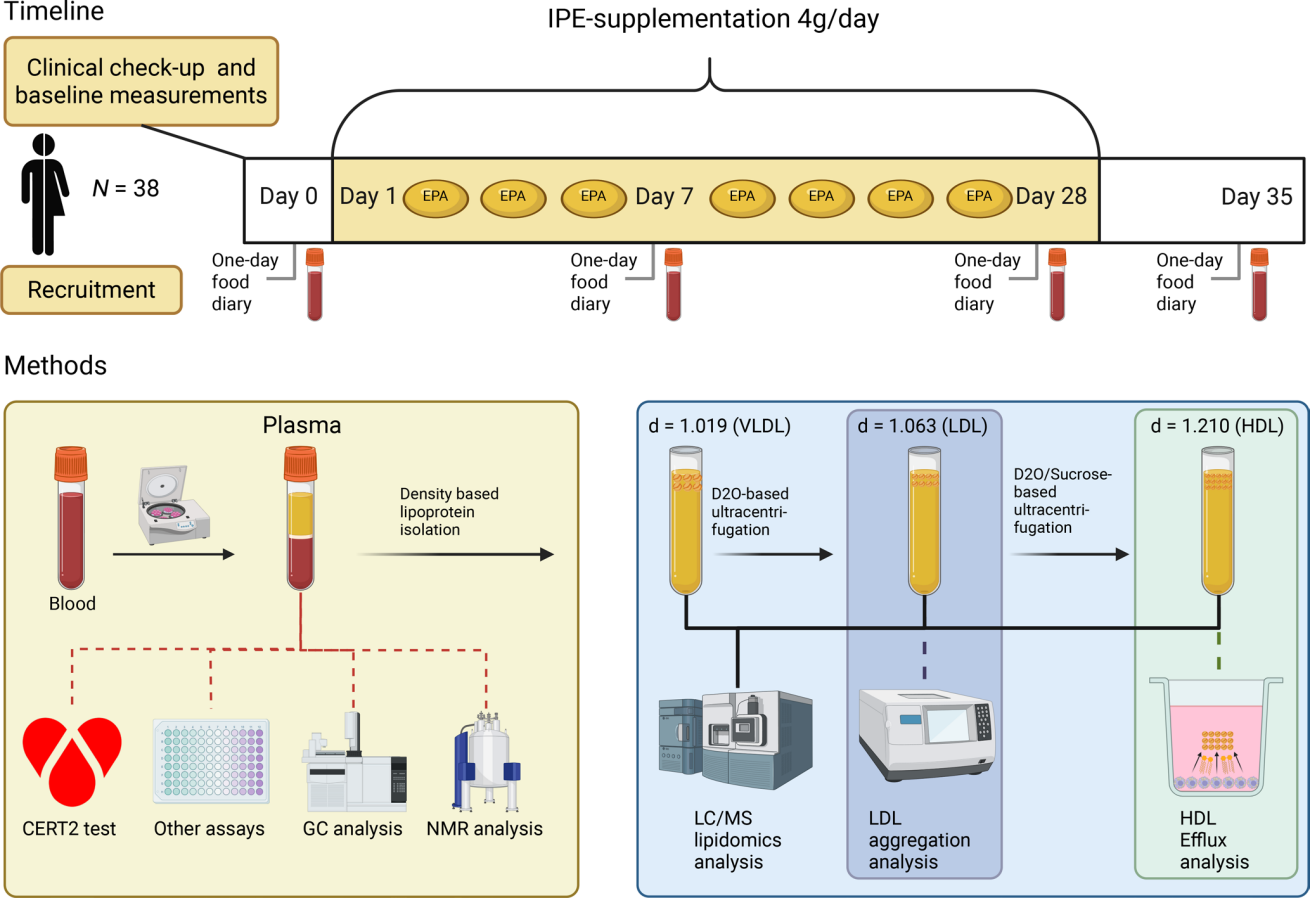
Study timeline and methods. Seventy-two participants (34 male and 38 female) were recruited. Clinical baseline measurements were taken before 28-day IPE supplementation. IPE, icosapent ethyl; CERT2, Coronary Event Risk Test 2; GC, gas chromatography; NMR, nuclear magnetic resonance (spectroscopy); LC/MS, liquid chromatography-mass spectrometry; VLDL, very low-density lipoprotein; LDL, low-density lipoprotein; HDL, high-density lipoprotein. Figure created with BioRender.com.

The study design is outlined in Figure 1, and the baseline characteristics of the study participants are shown in Table 1. All participants were normolipidemic and -glycemic men and women with a mean age of 30.

### IPE supplementation dramatically alters plasma fatty acid profile.

**Figure 2:**
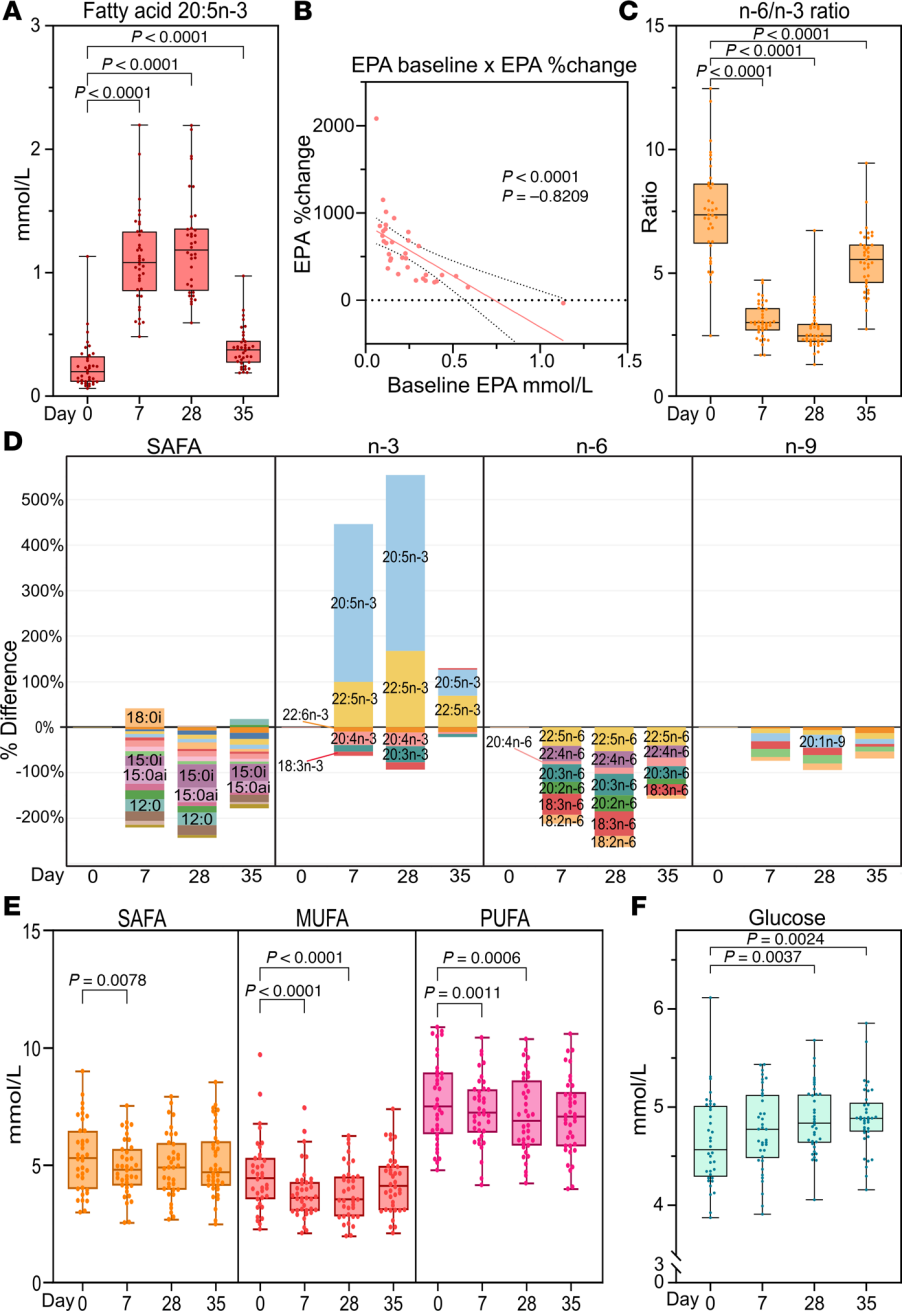
Effect of IPE supplementation on plasma fatty acids. Fasting plasma total fatty acids were measured by gas chromatography at baseline (day 0), at 7 and 28 days of IPE supplementation, and after washout on day 35. (**A**) Eicosapentaenoic acid (EPA, 20:5n-3) concentrations (*n* = 38). (**B**) Percentage change in plasma EPA between day 0 and day 28 plotted against baseline EPA concentrations (*n* = 34); a linear fit (red line) with 95% confidence intervals (dotted lines) are shown. A Spearman’s correlation analysis between the baseline concentration and EPA percentage change was conducted, with the correlation coefficient and *P* value shown. (**C**) Total fatty acid n-6/n-3 ratio (*n* = 38). (**D**) Relative changes in fatty acid mean concentrations versus baseline (*n* = 36). (**E**) Total plasma SAFA, MUFA, and PUFA concentrations (*n* = 38). (**F**) Plasma glucose concentrations (*n* = 37). For box plots, the boxes represent the 25th–75th percentile with a median bar, and whiskers represent the range with all individual values shown. Dots represent individual participant data points. Statistical significance was assessed using paired multiple *t* tests (Limma) with FDR correction.

Following IPE supplementation, the total circulating EPA concentration increased 4-fold within 7 days (Figure 2A). The additional 3-week IPE supplementation elevated EPA only marginally. During the 7-day washout period, EPA concentration dropped close to baseline levels. There was a significant person-to-person variation in the increase in plasma EPA concentration, which had a strong negative correlation with the baseline EPA concentration (Figure 2B).

The IPE preparation used in the study also contained vitamin D, resulting in increased serum levels of vitamin D (Supplemental Figure 1; supplemental material available online with this article; https://doi.org/10.1172/jci.insight.193637DS1), which, along with increased EPA concentrations, indicated compliance of the participants with the study design.

At baseline, n-3 PUFAs comprised 5% and n-6 PUFAs 38% of the total plasma fatty acids, and after 28 days their proportions were 13% and 32%, respectively (Supplemental Figure 2). Docosapentaenoic acid (DPA), the immediate elongation product of EPA, increased 3-fold after 28 days of IPE supplementation (Supplemental Figure 3 and Supplemental Table 1). Conversely, the concentrations of nearly all other fatty acids, including docosahexaenoic acid (DHA) and all n-6 PUFAs, decreased, resulting in a marked reduction of plasma n-6/n-3 ratio from 7.5 ± 2.1 at baseline to 2.6 ± 0.6 at day 28 (Figure 2, C and D). The decreases in several fatty acids, such as palmitic, oleic, and linoleic acid, led to a significant 10% ± 13% reduction in plasma total fatty acids as well as saturated fatty acids (SAFAs), monounsaturated fatty acids (MUFAs), and PUFAs (Figure 2E, Supplemental Figure 3, and Supplemental Table 1). During the 7-day washout period, EPA and DPA levels decreased, while all fatty acids that had been reduced by IPE supplementation, as well as the n-6/n-3 ratio, increased toward the baseline levels.

### IPE supplementation alters metabolic markers.

Next, we used NMR metabolomics to investigate how metabolic markers are affected by IPE supplementation. Plasma glucose levels increased significantly (Figure 2F) while pyruvate and lactate were significantly reduced after 28 days of IPE supplementation (Supplemental Table 2). Ketone bodies (acetoacetate, acetone, β-hydroxybutyrate) were significantly reduced after 7 days of IPE supplementation on average by 25%, 18%, and 32%, respectively (Supplemental Table 2). Several amino acids, most notably branched-chain amino acids and histidine, were significantly increased after IPE supplementation, while acetate was significantly reduced (Supplemental Table 2).

### Elevated EPA reduces CVD risk score and lipoprotein binding to proteoglycans.

**Figure 3:**
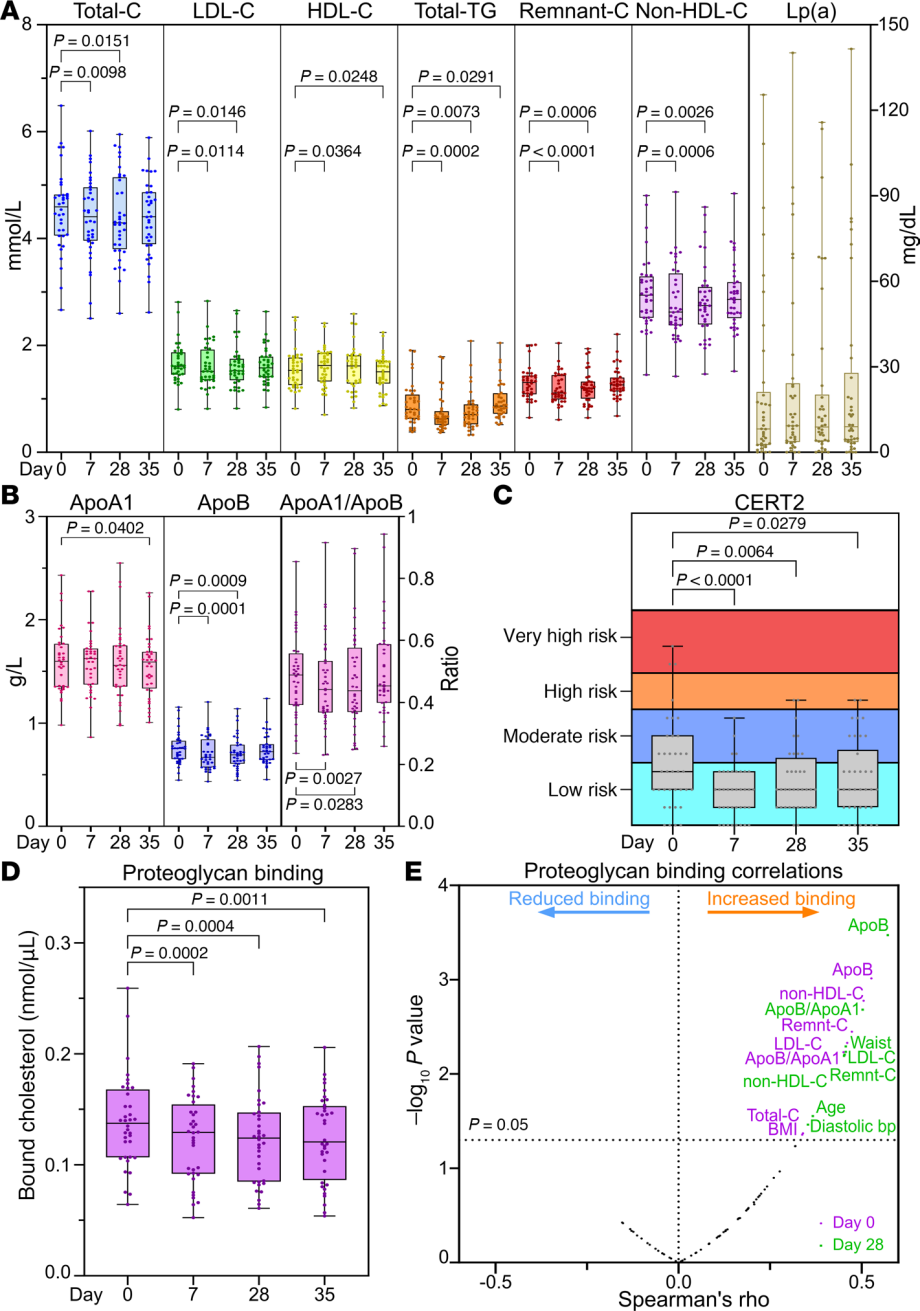
Effect of IPE supplementation on clinical plasma lipids, cardiovascular risk, and lipoprotein pro-atherogenic properties. (**A**) Fasting plasma clinical lipid markers measured by NMR spectroscopy at baseline, day 7, day 28, and after washout on day 35 (*n* = 36). “Lp(a)” indicates lipoprotein (a). (**B**) Plasma apoA1, apoB, and apoB/apoA1 ratio, determined by NMR spectroscopy (*n* = 36). (**C**) Coronary event risk test 2 (CERT2) was performed on plasma samples at all 4 time points (*n* = 38). The test categorizes individuals into a risk category, indicated by different colors in the graph. (**D**) The affinity of plasma lipoproteins for aortic proteoglycans was assayed ex vivo as described in the Methods (*n* = 36). (**E**) Spearman’s correlation analysis between the binding affinity of lipoproteins to aortic proteoglycans and clinical parameters and plasma lipid levels (*n* = 36). For box plots, the boxes represent the 25th–75th percentile with a median bar, and whiskers represent the range with all individual values shown. Statistical significance was assessed using paired multiple *t* tests (Limma) with FDR correction.

IPE supplementation significantly reduced plasma TG levels by 14% ± 28%, apoB by 6% ± 11%, and remnant cholesterol (lipoprotein-associated cholesterol, abbreviated hereafter by -C) by 8% ± 11% already after 7 days (Figure 3, A and B). The reduction in remnant-C resulted in a reduction in total cholesterol despite a statistically significant increase in HDL-C (Figure 3A). Additionally, non-HDL-C and LDL-C were reduced after the 7-day IPE supplementation (Figure 3A). All the observed changes returned toward their initial levels after the 7-day washout period. Lp(a) concentrations did not respond to IPE supplementation (Figure 3A).

CERT2 score (26–30) in our cohort of healthy normolipidemic individuals was low or moderate in most participants at baseline. Nevertheless, the risk scores decreased on average by 26% following 28 days of IPE supplementation (Figure 3C).

To assess whether IPE supplementation affects pro-atherogenic properties of lipoproteins, we assayed the binding of lipoproteins to human aortic proteoglycans and the aggregation propensity of LDL particles. These are markers of lipoprotein retention in the subendothelial extracellular matrix and the susceptibility of LDL to undergo modifications and aggregate during atherogenesis. IPE supplementation significantly decreased the amount of lipoprotein cholesterol bound to aortic proteoglycans from baseline to day 28 and remained reduced even after the washout period (Figure 3D). The proteoglycan binding positively correlated with both circulating and clinical risk markers of CVD (Figure 3E). IPE-induced change in LDL aggregation rate between day 0 and 28 correlated negatively with the baseline rate, indicating that LDL aggregation susceptibility increased in those with aggregation-resistant LDL at baseline but decreased in the individuals who had the most aggregation-prone LDL (Supplemental Figure 4).

We also determined the effect of IPE supplementation on reverse cholesterol transport, a key mechanism for combating cholesterol accumulation in the arterial wall. We did not observe any IPE-induced differences in the capacity of either plasma or isolated HDL particles to remove cholesterol from macrophages (Supplemental Figure 5).

### Decrease in atherogenic lipoprotein particles and their lipids due to IPE supplementation.

To determine in detail how lipoproteins and their subclasses, particle sizes, and particle and lipid concentrations were affected by the IPE supplementation, plasma samples were analyzed by NMR spectroscopy. Lipoprotein subclass descriptions and data are available in Supplemental Table 3.

**Figure 4:**
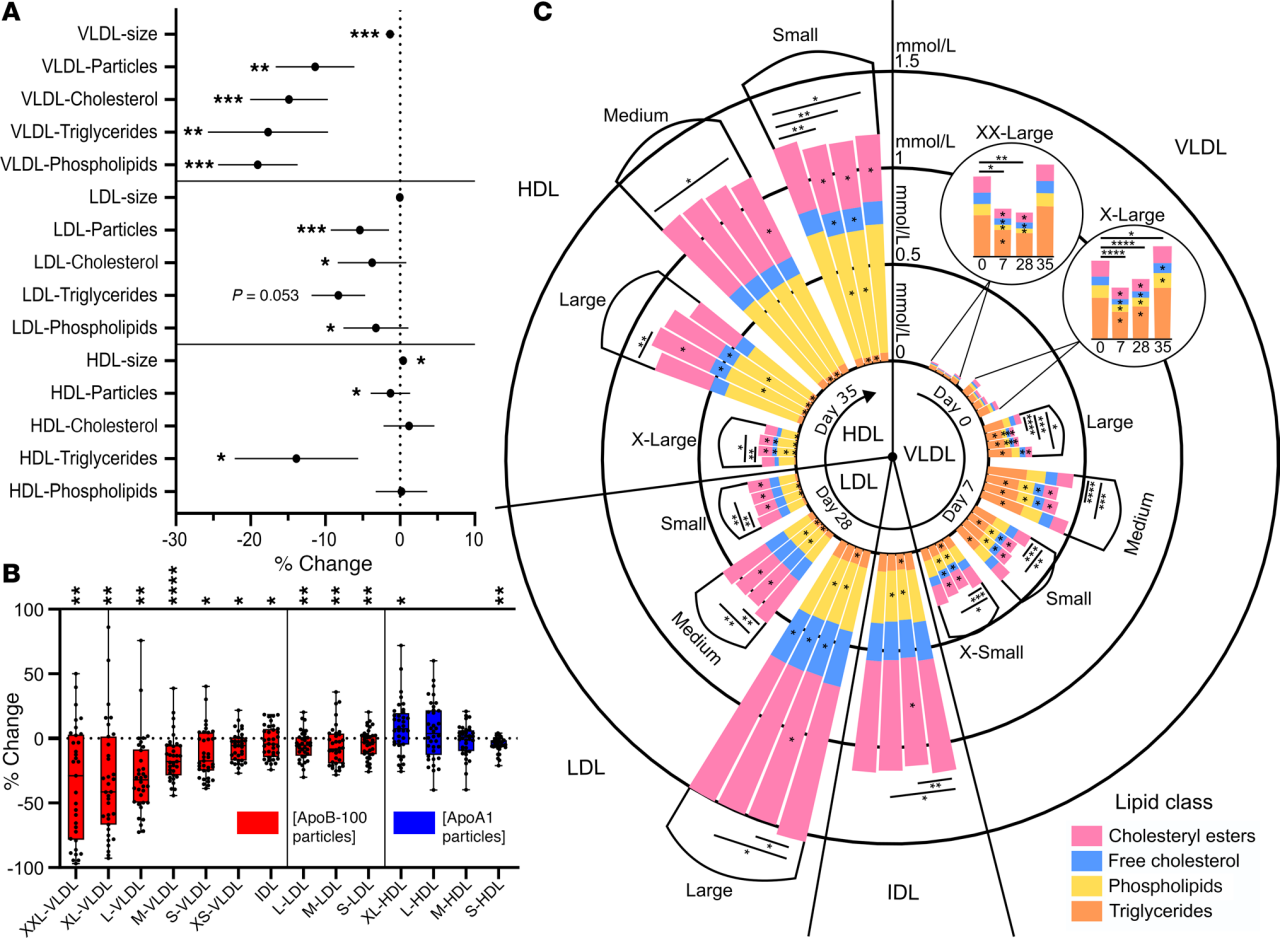
Effect of IPE supplementation on lipoprotein subclasses. Plasma concentrations, particle sizes, and lipid compositions of 14 lipoprotein subclasses were measured using NMR spectroscopy. (**A** and **B**) Percentage change in lipid and particle concentrations or particle sizes between baseline and day 28. In **A**, data are means ± 95% confidence interval. In **B** box plots show median and interquartile range with individual data points. Significant changes are marked *P < 0.05, **P < 0.01, ***P < 0.001, and ****P < 0.0001. (**C**) Circular stacked bar plot of lipoprotein subclass lipid compositions at baseline, day 7, day 28, and day 35. Each bar shows total lipid concentration of a lipoprotein subclass with colors for lipid classes. The time points are shown in a clockwise rotation order for each lipoprotein subclass, organized based on their size from largest to smallest. Significant changes in lipid abundances are indicated by * within bars. Total lipid differences are indicated on top; *P < 0.05, **P < 0.01, ***P < 0.001, and ****P < 0.0001. Statistical significance was assessed by paired multiple *t* tests (Limma) with FDR correction. *N* = 36 for all panels.

The number of circulating VLDL particles decreased by 9% ± 24% from baseline to day 28 of IPE supplementation (Figure 4A). This reduction in particle number was accompanied by reductions in VLDL-C, VLDL-phospholipids (PL), VLDL-TG, and VLDL size (37.6 nm vs. 37.1 nm) (Figure 4A). The largest relative changes in particle numbers and lipids carried in those particles were observed in the largest lipoprotein subclasses. The particle number of XXL-VLDL (size > 75 nm) decreased by 35% in contrast with XS-VLDL (mean size 31 nm), which decreased by 6% (Figure 4B). PLs, free cholesterol (FC), and cholesteryl esters (CE) were reduced in all VLDL subclasses, while TGs were reduced in XL-, L-, and M-VLDL after 28 days of IPE supplementation (Figure 4C).

The total number of circulating LDL particles, and hence total LDL lipid, LDL-C, and LDL-PL, decreased slightly, with no change in the average particle size (Figure 4A). Statistically significant reductions of 2%–7% were observed in total lipid and particle concentrations in all 3 LDL subclasses (Figure 4B). Additionally, FC was reduced in L-LDL, while CE and TG were reduced in M- and S-LDL and PLs in IDL, L-, and M-LDL after 28 days of IPE supplementation (Figure 4C).

The total number of circulating HDL particles reduced after IPE supplementation (Figure 4A). The number of S-HDL particles decreased, and the number of XL-HDL particles increased (Figure 4B), leading to an overall increase in HDL particle size. HDL-C increased at day 7 but did not differ from baseline at day 28, unlike HDL-TG, which decreased in all 4 HDL subclasses after 28 days of IPE supplementation (Figure 4C). The shift toward larger HDL particles resulted from increases in FC, CE, and PL carried in L-HDL and decreases in S-HDL.

With respect to relative proportions of lipids within lipoproteins, a reduction in TG content was observed only in HDL, where it decreased in all 4 subclasses. The proportion of FC+CE was reduced in M-LDL and S-LDL, while PLs increased in S-LDL, M-LDL, and IDL. VLDL particles showed a reduction in the relative FC+CE content only in S-VLDL (Supplemental Table 3).

### IPE supplementation decreases nonesterified fatty acids and affects regulators of LPL.

To investigate how IPE supplementation might cause the reductions in lipoprotein particle numbers, we analyzed the concentration of circulating nonesterified fatty acids (NEFAs), which contribute to the hepatic lipid storages, a crucial factor in VLDL production (31). The NEFAs decreased significantly by 15% following IPE supplementation, and the decrease persisted after the washout period (Supplemental Figure 6A).

Omega-3 fatty acids can directly upregulate LPL (25), but its enzymatic activity is mainly controlled posttranslationally via inhibitors such as apoC-III and angiopoietin-like 3 protein (ANGPTL3). ANGPTL3 was significantly reduced after IPE supplementation (Supplemental Figure 6B). ApoC-III decreased after 7 days of IPE supplementation and remained significantly lower after washout (Supplemental Figure 6C). ApoC-II, which is a cofactor of LPL, did not change following IPE supplementation (Supplemental Figure 6D).

### Impact of IPE supplementation on lipoprotein lipidomes.

**Figure 5:**
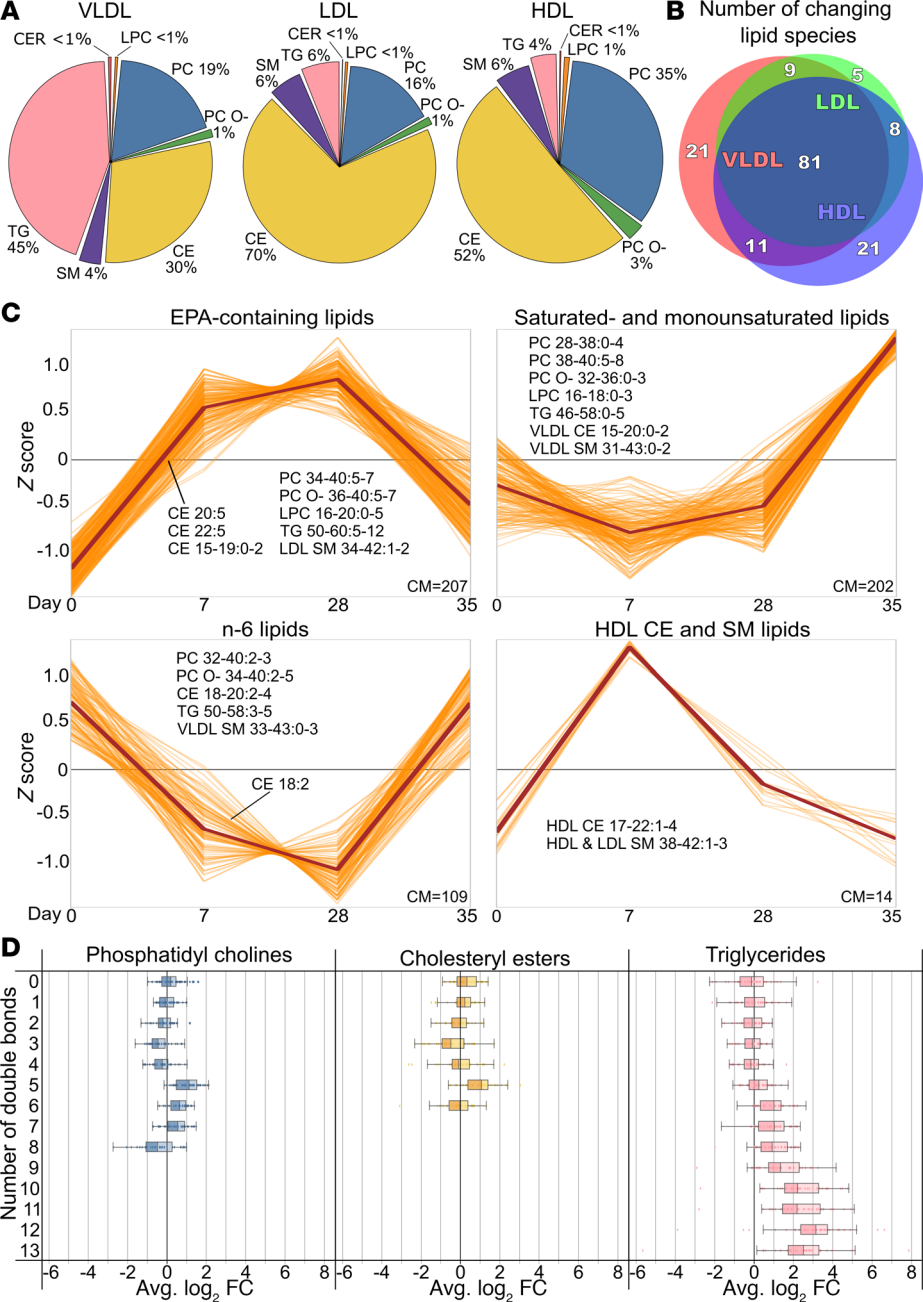
Impact of IPE supplementation on lipoprotein lipidome composition and dynamics. Lipoprotein fractions were isolated by ultracentrifugation, and their lipids were analyzed by LC/MS. (**A**) Pie charts show lipid class compositions of isolated lipoproteins at baseline (day 0). (**B**) Venn diagram displays significantly changing lipid species in each fraction after 28 days. (**C**) Time series clustering of significantly changing HDL, LDL, and VLDL lipids; orange lines represent individual species and brown lines, cluster medians; and cluster member counts (CM) are shown. Only species with cluster membership of >0.5 are included. Panels are named based on typical cluster members, which are listed in the panels. Lipid annotation: class, acyl carbon range, and double bond range. (**D**) Change in LDL lipid unsaturation from baseline to day 28, grouped by double bond count. Box plots show mean log_2_ fold-changes with medians, 1.5 interquartile ranges, and individual data points. *N* = 29 for HDL, 37 for LDL, and 38 for VLDL. Venn diagram implemented with DeepVenn, © Tim Hulsen, https://www.deepvenn.com

VLDL, LDL, and HDL were isolated by ultracentrifugation and their lipids then analyzed by LC/MS. The lipid class compositions of the isolated lipoproteins at baseline were as expected (Figure 5A and Supplemental Table 4, A and B). The relative lipid class proportions were generally resistant to IPE supplementation (Supplemental Table 4B). However, the relative proportion of VLDL-TG increased significantly from 45% to 48%, while that of VLDL-CE decreased significantly from 30% to 26% over 28-day IPE supplementation.

LC/MS revealed a 15% increase in the HDL lipid-to-protein, consistent with the increased HDL particle size observed in NMR analyses (cf. Figure 4). The increased lipid-to-protein was due to elevated HDL-CE, HDL-diacylphosphatidylcholine (PC), HDL-lysophosphatidylcholine (LPC), and HDL-sphingomyelin (SM) (Supplemental Table 4A). No changes in total lipid-to-protein ratio were observed for VLDL or LDL particles, but VLDL-ceramide (Cer) decreased significantly.

At the individual lipid species level, drastic changes were observed across all lipoprotein fractions and lipid classes. After 28 days of IPE supplementation, out of the 315 detected lipid species, significant changes were noted in 122 species in VLDL, 103 in LDL, and 121 in HDL (Figure 5B). The most significant changes in each lipoprotein fraction are presented in Supplemental Figure 7. All individual lipid species abundances and their changes are detailed in Supplemental Table 5.

Feature-based variance-sensitive clustering of all lipid abundances across all lipoprotein classes identified 4 distinct clusters, each showing unique lipid species changes across the 3 lipoprotein fractions (Figure 5C). The clustering analysis indicated that the most substantial changes were observed in EPA-containing lipids, which showed a significant increase, and in n-6 PUFA–containing lipids, which demonstrated a notable decrease. Taken together, significant increases in EPA-containing CE, PC, and TG species, as well as reductions in saturated, monounsaturated, and n-6 PUFA species, led to a clear shift toward a higher degree of unsaturation following IPE supplementation (Figure 5D and Supplemental Figure 8).

### The distribution of EPA in the circulating lipoproteins.

**Figure 6:**
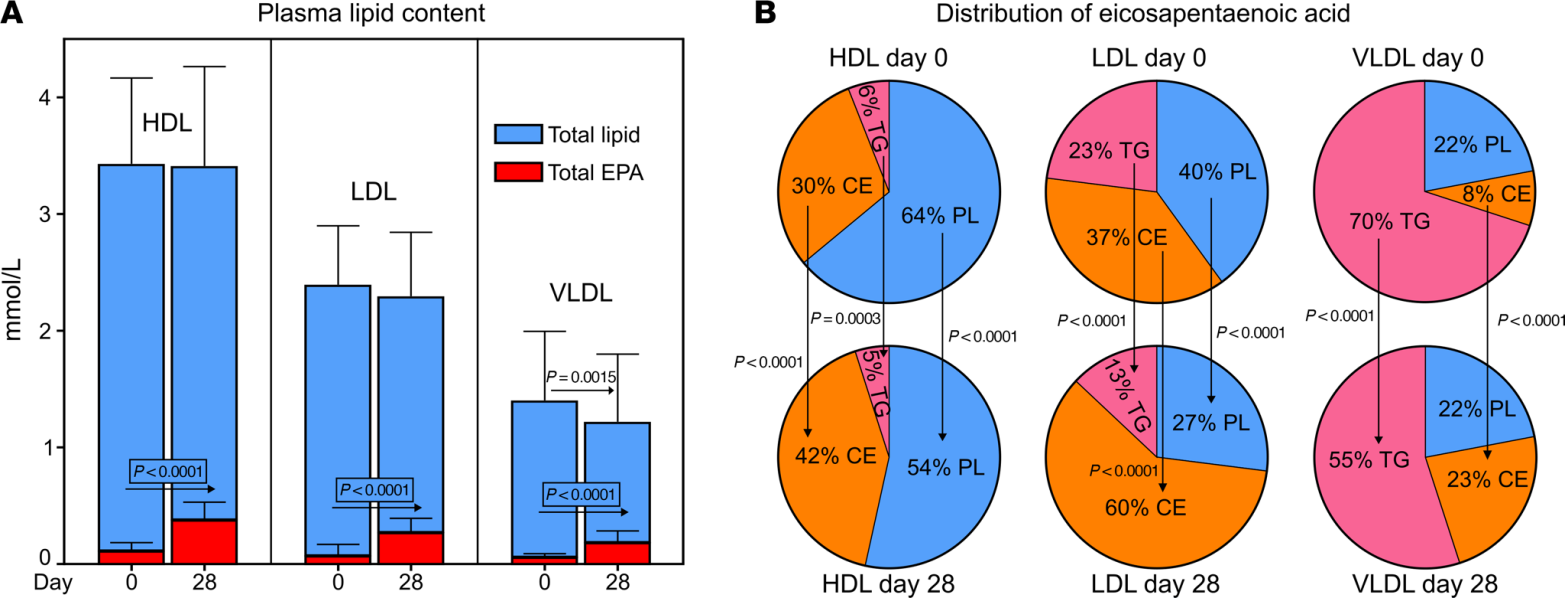
Impact of IPE supplementation on EPA distribution in plasma and lipoproteins. (**A**) The distribution of circulating total lipids and EPA in lipoproteins. Total lipid concentrations (blue bars) in HDL, LDL, and VLDL were determined by NMR spectroscopy, and EPA-containing lipid fractions (red bars) were assessed by LC/MS (see Supplemental Methods). Data are means ± SD. (**B**) EPA distribution across lipid classes in HDL, LDL, and VLDL at baseline and after 28 days. Lipid species were analyzed by LC/MS; pie charts show the EPA-containing proportions of each lipid class. *N* = 29 for HDL, 37 for LDL, and 38 for VLDL. *P* values were determined using 1-way ANOVA with mixed effects modeling and FDR correction.

The distribution of EPA among lipoprotein fractions mirrored the overall distribution of lipids (Figure 6A and Supplemental Figure 9). The majority of EPA was carried in HDL particles, while VLDL particles contained a higher proportion of EPA than of total lipid. IPE supplementation significantly shifted the distribution of EPA-containing lipid species from the VLDL pool toward LDL (Supplemental Figure 9).

The distribution of EPA between lipid classes in each lipoprotein fraction is shown in Figure 6B. IPE supplementation significantly shifted the distribution of EPA from PL and TG toward CE.

We estimated the number of lipid molecules in a single lipoprotein particle and the number of EPA molecules it carried (Table 2). After IPE supplementation, the number of EPA-containing lipid species per particle increased from 8 to 15 in HDL, from 60 to 220 in LDL, and from 500 to 1,500 in VLDL.

### Uncovering of individual lipoprotein lipid fingerprints.

**Figure 7:**
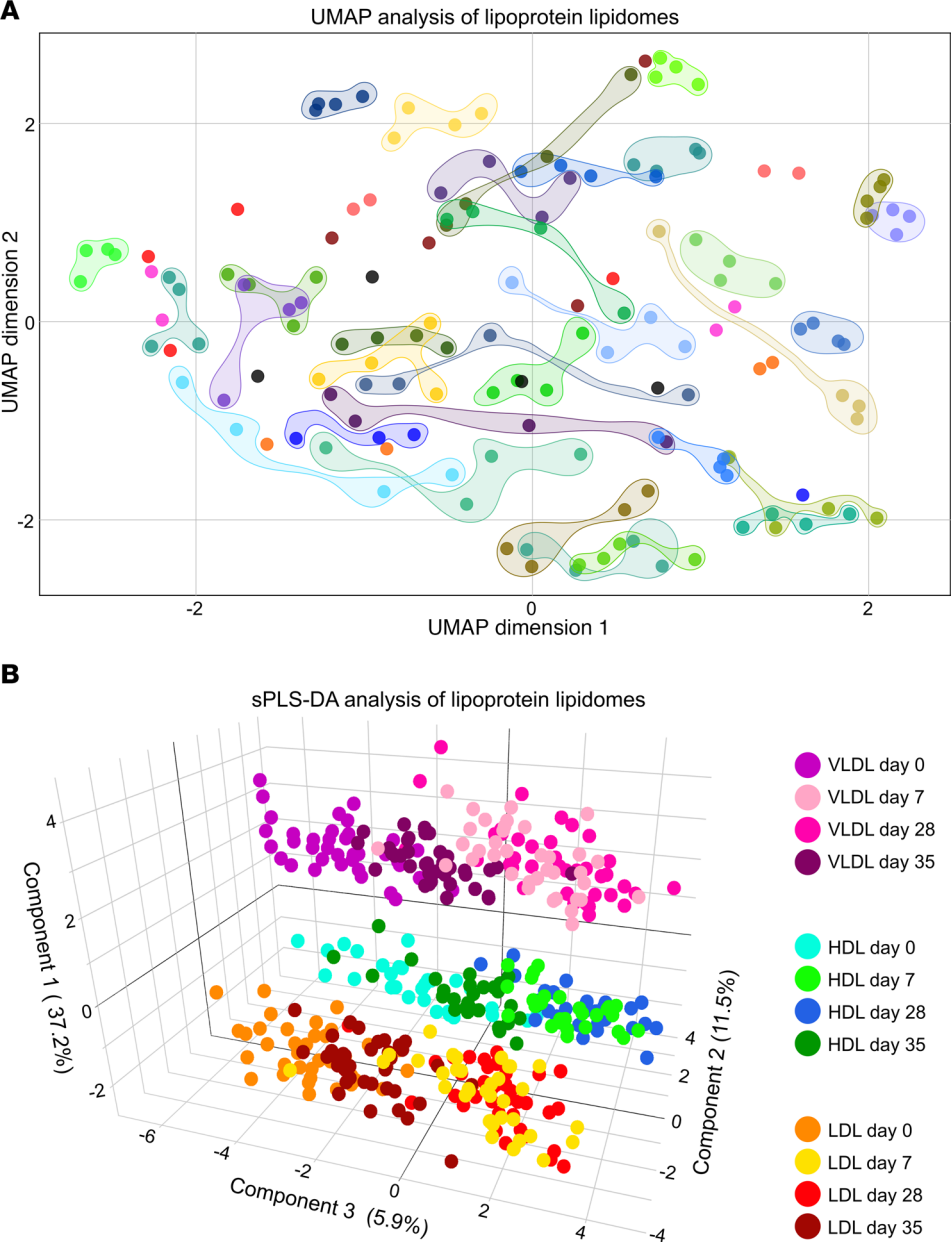
Global analysis of IPE supplementation effects on lipoprotein lipidomes. (**A**) UMAP of combined HDL, LDL, and VLDL lipidomes (see Supplemental Methods). Each point represents an individual sample at day 0, day 7, day 28, or day 35, colored by participant; circled when clustering is obvious. (**B**) sPLS-DA of HDL, LDL, and VLDL lipidomes. Each point represents a sample from an individual; colors indicate lipoprotein class and time point (see color legend). Variance explained by each component is shown on the axes.

Although IPE supplementation altered the lipoprotein lipidomes, principal component analysis (PCA) did not show strong IPE-related clustering (Supplemental Figure 10). Linear mixed model analyses of the principal components showed that random (subject) effects dominated over fixed effects (time), indicating that intersubject variability accounted for most of the variance (Supplemental Table 6). To quantify this, we compared Euclidean distances between observations. Distances were longest between different subjects, slightly shorter between time points, and approximately 2-fold shorter within subjects (Supplemental Figure 11), reflecting strong individual lipid fingerprints. Hierarchical clustering supported this, with observations from each subject clustering together or on adjacent branches (Supplemental Figure 12). Uniform manifold approximation and projection (UMAP) analysis also showed distinct within-subject clustering in 2 dimensions (Figure 7A).

Supervised sparse partial least squares regression-discriminant analysis (sPLS-DA) revealed clear separation of VLDL, LDL, and HDL lipidomes along the first 2 components (Figure 7B). The third component showed changes between day 0 and day 7, with day 7 and 28 clustering together. Day 35 partially overlapped with both day 0 and day 7/28 groups, indicating that IPE-induced changes occurred predominantly during the first 7 days and partially reverted during washout.

### Metabolites and clinical parameters associated with pro-atherogenic properties of lipoproteins.

**Figure 8:**
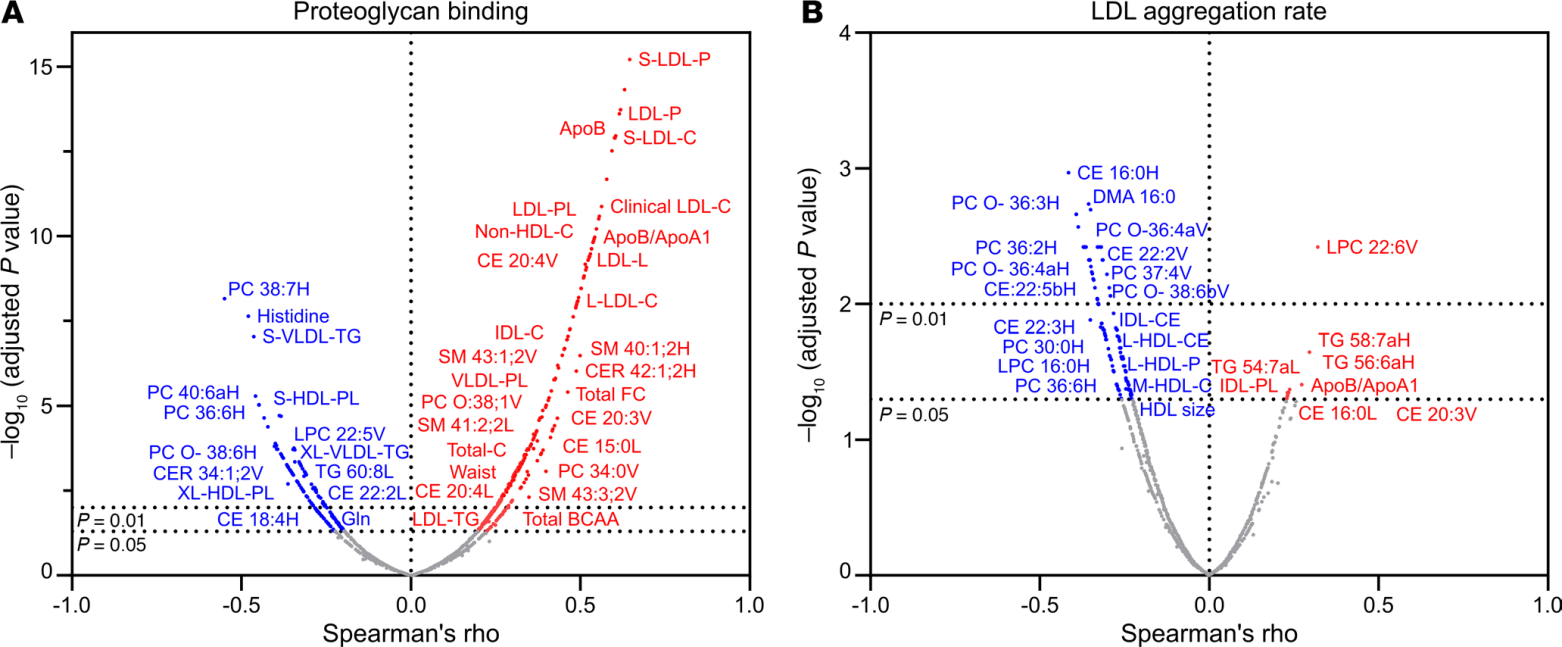
Correlation analysis of factors influencing lipoprotein binding to proteoglycans and LDL aggregation. Binding affinity and LDL aggregation propensity were assessed as described in the Methods. Biomarker and lipid abundances in plasma and isolated lipoproteins were measured by NMR spectroscopy and LC/MS across 4 time points (day 0, day 7, day 28, and day 35). (**A**) Volcano plot of Spearman’s correlations between metabolite/biomarker abundances and proteoglycan binding; significant correlations are colored (blue = negative, red = positive), with selected metabolites/biomarkers labeled. (**B**) Volcano plot of correlations with LDL aggregation rate, with color coding as in **A**. Lipid species names ending in H, L, or V indicate association with HDL, LDL, or VLDL. *N* = 29 for HDL, 37 for LDL, and 38 for VLDL.

Next, we investigated how the individual metabolic profiles influence the pro-atherogenic properties of the lipoproteins. Spearman’s correlation analysis between all measured biomarkers, including clinical measurements, and proteoglycan binding (Figure 8A) or LDL aggregation rate (Figure 8B) revealed hundreds of metabolites and clinical parameters that significantly correlated with these atherogenic properties (Supplemental Table 7). As expected, the number of circulating apoB-containing lipoproteins had the strongest association. In addition, LDL-C, remnant-C, and total-C associated with increased proteoglycan binding (Figure 8A). Almost all individual CE and SM lipid species were positively associated with increased proteoglycan binding, similarly in both LDL and VLDL. In contrast, several of the EPA-containing HDL-PC species, and plasma amino acids histidine and glutamine, were negatively correlated with proteoglycan binding.

Even though plasma was not directly involved in the LDL aggregation assay, plasma components such as lipoproteins and their lipid exchange dynamics with LDL are crucial. Changes in these components can affect LDL lipid composition, which in turn impacts LDL aggregation. As such, we found that increased LDL aggregation rate was associated with apoB/apoA1 ratio, CE 16:0 in LDL, and CE 20:3 and LPC 22:6 in VLDL, in addition to a few TG species (Figure 8B). HDL-C, HDL size, L-HDL-C, and HDL particle number, as well as several PC, PC O- (ether PC), LPC, and CE species were correlated with reduced LDL aggregation rate (Figure 8B).

**Figure 9:**
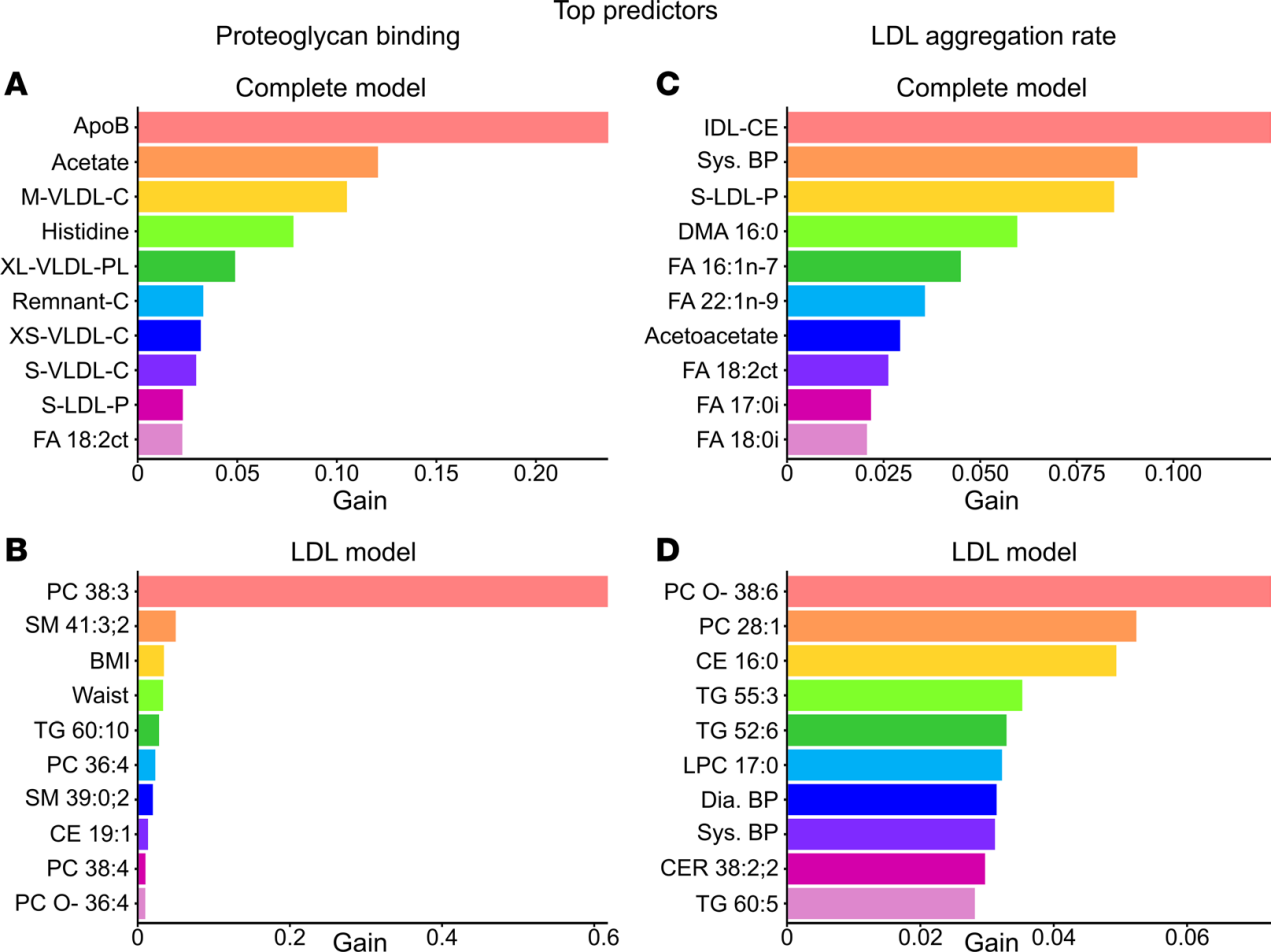
Machine learning analysis of predictors for lipoprotein pro-atherogenic properties. Proteoglycan binding and LDL aggregation were assessed as described in Methods. Biomarker and lipid abundances were measured by NMR spectroscopy and LC/MS. XGBoost models evaluated contributions of clinical, metabolomic, and lipidomic features across all 4 time points. Two different models were constructed: complete model (all data) and LDL model (LDL lipidome + clinical data). (**A** and **B**) Top 10 predictors of proteoglycan binding. (**C** and **D**) Top 10 predictors of LDL aggregation. Bars represent predictor contributions (gain) in each model.

To further understand the risk markers involved in proteoglycan binding and LDL aggregation, we utilized XGBoost machine learning models to highlight associated metabolites and clinical parameters. Models were constructed using plasma metabolites, lipoprotein lipidomes, and clinical parameters (complete model, Figure 9, A and C) or LDL lipids and clinical parameters (LDL model, Figure 9, B and D).

The complete model identified apoB and several associated cholesterol variables, along with acetate, histidine, and fatty acid 18:2n-6, as the best predictors of proteoglycan binding (Figure 9A). In the LDL model, a single very strong predictor, LDL-PC 38:3, emerged (Figure 9B). Interestingly, LDL-PC 38:3 was significantly reduced by 60% after IPE supplementation (cf. Supplemental Table 5). Waist circumference, age, and BMI were identified by machine learning models and Spearman’s correlation analysis as factors associated with increased proteoglycan binding.

Regarding LDL aggregation, the complete model identified fatty acids with 16–18 carbons and 0–2 double bonds, along with systolic blood pressure, as the best predictors (Figure 9C). In the LDL model, PC O- 38:6 and various lipid species with a low degree of unsaturation, along with blood pressure, were identified as the strongest predictors (Figure 9D).

## Discussion

Our findings in healthy normolipidemic individuals suggest that IPE supplementation alters systemic metabolism to favor fatty acid oxidation over glucose and leads to reduction in apoB lipoprotein secretion, plasma lipids, and lipoprotein retention propensity. These changes were evident after 7 days and attenuated after a 7-day washout.

Consistent with previous studies, daily IPE supplementation resulted in a 4-fold increase in circulating EPA, accompanied by increased DPA but no increase in DHA, suggesting limited conversion, as also previously noted (32). This shift was accompanied by reductions in SAFA, MUFA, and n-6 PUFA, resulting in a substantially lower n-6/n-3 ratio and increased overall unsaturation, changes associated with reduced inflammation and CVD risk (33, 34). The observed improvement in the CERT2 risk score further supports these systemic benefits.

Sustained high EPA levels throughout the 28-day supplementation and their decrease after a 7-day washout indicate a strong but reversible effect of IPE. Consistently, kinetic studies indicate rapid EPA clearance from circulation (35, 36). Clinical trials often imply a longer time frame for sustained EPA levels with IPE supplementation (1, 2). Although it is possible that EPA persists particularly in the adipose tissue and potentially induces long-term effects, our results suggest that adherence to the supplementation is crucial for sustained benefits. On the other hand, the rapid clearance of EPA enables quick attenuation of potential IPE-induced side effects.

Plasma total lipid, TG, and cholesterol levels were significantly reduced during supplementation, aligning with previous findings in hyperlipidemic patients (1, 2). The reduction was mainly due to depletion of large TG-rich apoB particles, likely aligning with decreased hepatic VLDL secretion. Circulating NEFA levels declined significantly, suggesting reduced adipose lipolysis or enhanced clearance by oxidative tissues, thereby limiting the hepatic TG pool and hence VLDL secretion (37, 38).

The reduction in the number and size of TG-rich apoB lipoproteins also reflects increased LPL activity, supported by decreased plasma levels of LPL inhibitors ANGPTL3 and apoC-III. Previous studies have suggested increased LPL activity after n-3 supplementation (39–41), partly due to reduced apoC-III (42); however, the mechanism by which IPE impacts ANGPTL3 remain unknown. Decreased levels of SAFA and MUFA, which are preferred LPL substrates, further support increased lipid turnover.

IPE supplementation appears to induce systemic metabolic remodeling, as indicated by decreased plasma lactate and increased fasting glucose, suggesting improved aerobic metabolism and reduced reliance on anaerobic glycolysis. Concurrent increases in acetate and reductions in NEFAs are consistent with enhanced fatty acid β-oxidation, likely mediated by activation of PPARα and PPARβ/δ (43, 44). These PPAR isoforms upregulate key oxidative genes (e.g., CPT1A, ACOX1, ACADL, PDK4) while suppressing lipogenesis via inhibition of SREBP-1c (45). In parallel, modulation of PPARγ activity in adipose tissue may contribute to reduced adipose tissue lipogenesis and improved insulin sensitivity. The observed reductions in canonical de novo lipogenesis (DNL) markers (fatty acid 14:0, 16:0, 16:1, and 18:1) support suppression of lipogenesis. Consistent with these results, a previous study found that hepatic DNL is suppressed and fat oxidation is increased by omega-3 fatty acids at the expense of glucose metabolism (46).

The observed reductions in DNL-associated fatty acids may have clinical relevance, as elevated circulating levels of these lipids have been linked to increased risk of all-cause mortality (47), cardiometabolic disease (48), and incident type 2 diabetes independent of insulin sensitivity (49). These associations underscore the potential of targeting DNL and its lipid products as a means to reduce long-term disease risk and suggest that the metabolic effects of IPE may extend beyond lipid profile modulation.

Our data showed an unexpected reduction in fasting plasma ketone bodies, as increased hepatic fatty acid oxidation is typically associated with elevated ketogenesis (50). However, consistent with the present findings, fish oil supplementation has been shown to decrease circulating β-hydroxybutyrate (46, 51). This could be due to suppression of ketogenic enzymes, as EPA is associated with improved insulin sensitivity and sustained hepatic insulin function (52). Additionally, the observed increase in circulating ketogenic amino acids suggests that reduced ketone bodies may result from inhibition of hepatic amino acid catabolism.

IPE was rapidly incorporated into the lipidomes of VLDL, LDL, and HDL, with pronounced overrepresentation in VLDL. EPA content increased from 3%–5% at baseline to 12%–16% at day 28 and returned to baseline during washout. One-third of individual lipid species in VLDL, LDL, and HDL were significantly impacted by IPE supplementation (Figure 5 and Supplemental Table 5). EPA enrichment occurred across lipid classes, especially in CE at the expense of PL and TG. The largest absolute pool of EPA was in LDL-CE, accounting for 3% of all circulating lipids. Since hepatic incorporation of EPA into CE is known to be inefficient (53), the observed enrichment likely reflects lipid remodeling in the circulation. This remodeling is mediated by lecithin:cholesterol acyltransferase (LCAT), which catalyses CE formation in HDL, and by cholesteryl ester transfer protein (CETP), which facilitates the exchange of HDL-CE for TG in VLDL and LDL. The observed increase in both HDL-CE and HDL-LPC observed after IPE supplementation supports enhanced LCAT activity. Meanwhile, CETP activity, previously shown to increase with n-3 PUFA supplementation (54), likely contributes to the redistribution of EPA-enriched CE from HDL to LDL and VLDL.

Despite the extensive lipidome remodeling induced by IPE, individual participants’ lipidomic profiles remained distinct. Unsupervised clustering revealed strong person-specific patterns, indicating the persistence of unique lipidomic fingerprints even after a major metabolic intervention. This suggests that personal lipoprotein lipidomes are shaped by inherent, possibly unmodifiable factors. Indeed, two-thirds of lipid species within VLDL, LDL, and HDL remained unaffected by IPE supplementation. Notably, there was considerable interindividual variability in EPA incorporation into plasma lipids, with observed increases ranging from 1- to 20-fold, consistent with previous studies (55). The increase was inversely correlated with baseline EPA level, suggesting a saturation threshold beyond which additional EPA is shunted toward β-oxidation rather than incorporation into lipoproteins.

IPE supplementation markedly increased the number of EPA-containing lipid molecules in apoB-containing lipoproteins. On average, EPA content rose by approximately 150 molecules per LDL particle and 950 molecules per VLDL particle, corresponding to relative increases from 2% to 10% and from 4% to 13%, respectively. In parallel, the observed reduction in SAFA contributed to a shift toward a less inflammatory lipid cargo. This compositional change may lower the atherogenic potential of these lipoproteins by reducing their capacity to promote vascular inflammation and plaque development.

Although participants exhibited no signs of overt inflammation, the lipidomic shifts observed following IPE supplementation suggest the potential for systemic immunomodulation. Incorporation of EPA into circulating lipoproteins may alter their interactions with vascular and immune cells — not only by reducing lipoprotein oxidation and aggregation but also by modifying the lipid cargo delivered to the endothelium and immune cells (56).

Once incorporated into cellular membranes, EPA displaces arachidonic acid (AA), leading to changes in membrane composition and lipid raft organization (56). This disrupts pro-inflammatory receptor signaling pathways, including those mediated by TLR4 and NF-κB, resulting in reduced transcription of inflammatory cytokines such as IL-6, TNF-α, and monocyte chemoattractant protein-1 in monocytes and endothelial cells (57–59). Additionally, EPA competes with AA for cyclooxygenase and lipoxygenase enzymes, shifting eicosanoid production toward less inflammatory mediators, such as prostaglandin E_3_ and leukotriene B_5_ (57, 59).

Importantly, EPA also serves as a precursor for specialized pro-resolving mediators, including resolvin E1, which acts via ChemR23 receptors on macrophages and vascular cells to enhance efferocytosis, suppress cytokine release, and limit uptake of oxidized LDL. These actions support resolution of inflammation and promote plaque stability. Given that ChemR23 also binds chemerin, an adipokine involved in adipose and hepatic inflammation, elevated EPA may influence receptor–ligand dynamics in metabolic tissues, contributing to broader immunometabolic regulation (7, 60).

EPA also exerts antithrombotic effects by competing with AA for platelet COX-1, reducing thromboxane A2 (TXA2) synthesis while increasing formation of the less active TXA3, thereby attenuating platelet aggregation and vasoconstriction (61). Furthermore, EPA promotes endothelial production of prostacyclin and downregulates platelet adhesion molecules, limiting platelet–leukocyte aggregate formation and vascular inflammation (61). Together, these mechanisms contribute to the antiinflammatory, antithrombotic, and cardioprotective profile of EPA.

IPE-induced changes in apoB-containing lipoproteins led to a marked reduction in their binding affinity to vascular proteoglycans. While apoB concentration itself was the strongest predictor of proteoglycan binding, specific lipid species also contributed significantly. Among these, cholesterol, but not TGs, carried within apoB particles showed a strong positive association with binding affinity. Of particular interest, the phosphatidylcholine species LDL-PC 38:3 was significantly reduced following IPE supplementation and showed a strong positive association with proteoglycan binding. This lipid has previously been linked to CVD risk (62), and future studies could explore its mechanistic role in lipoprotein–proteoglycan interactions and LDL aggregation using targeted experimental models. In contrast, EPA-containing lipid species were inversely associated with proteoglycan binding, potentially reflecting EPA-induced changes in apoB conformation that reduce its affinity for vascular matrix components (63, 64). We also identified negative correlations between proteoglycan binding and circulating levels of histidine and glutamine. These findings align with previous studies linking these amino acids to improved vascular health and reduced atherogenic risk (65, 66). Taken together, the IPE-induced reduction and compositional remodeling of apoB lipoproteins — and the resulting decrease in their proteoglycan retention — may help explain the previously reported reduction in cardiovascular events following IPE supplementation.

LDL aggregation susceptibility has been linked to a high SM-to-PC ratio and future CVD risk (11, 13). LDL aggregation susceptibility was unchanged overall but decreased in individuals with aggregation-prone LDL and increased in those with aggregation-resistant LDL. As IPE did not alter LDL-SM levels, these individual changes likely reflect baseline lipid composition. Such heterogeneity highlights the importance of personalized lipidomic responses.

Finally, HDL lipid remodeling following IPE supplementation resulted in a notable increase in EPA content, rising from 4% to 7%. This was accompanied by an increase in particle size and cholesterol content, along with a reduction in TG levels. These features are generally considered cardioprotective (67). Despite these favorable compositional changes, HDL-C uptake capacity from macrophages remained unchanged in our normolipidemic cohort. This suggests that enrichment of HDL with EPA does not necessarily translate into enhanced HDL function in metabolically healthy individuals. In contrast, previous studies have reported that EPA supplementation can improve HDL-mediated cholesterol uptake in individuals with dyslipidemia (68), indicating that the functional impact of HDL-EPA enrichment may depend on baseline metabolic status.

Lipoproteins up to 70 nm in diameter can penetrate the arterial intima, where a subset becomes retained by binding to arterial proteoglycans — a key initiating event in atherogenesis (6). IPE supplementation appears to reduce this atherogenic potential through multiple mechanisms: by lowering the number of circulating apoB-containing lipoprotein particles, decreasing their cholesterol content, and reducing their affinity for vascular proteoglycans. As a result, less lipid is likely to accumulate and be retained within the arterial wall following supplementation.

Beyond lowering lipid accumulation, IPE also profoundly remodels the lipid cargo of the lipoproteins that do become entrapped. This altered composition may actively limit atherosclerotic plaque development, particularly by reducing lipid volume and vascular inflammation within plaques (69). These changes are consistent with the formation of more stable, less rupture-prone plaques (70, 71), further contributing to the cardioprotective effects of IPE.

### Major limitations.

This study’s small sample size, recruitment of ethnic Finnish individuals, and focus on normolipidemic volunteers may limit its generalizability to broader populations, including those with cardiovascular conditions or dyslipidemia. Additionally, the IPE preparation contained vitamin D_3_, which can influence plasma lipoprotein profiles (72, 73). However, vitamin D kinetics differ from most other parameters, as its levels rise from baseline to week 1 and further to week 4 without decreasing during the washout period, whereas most other parameters peak at week 1 and return to baseline after washout. The study’s short duration also prevents assessment of IPE’s long-term effects on lipoprotein profiles and cardiovascular outcomes. Last, the mechanisms underlying the observed lipid changes and their relation to CVD risk remain unexplored, warranting further research.

## Methods

### Sex as a biological variable

The study included both male and female participants; however, sex-based differences were not evaluated.

### Study design and participants

This single-group, open-label study was conducted at the Wihuri Research Institute (Helsinki, Finland) between autumn 2019 and winter 2020. The study lasted 35 days and included 28 days of IPE supplementation followed by a 7-day washout period. Blood samples were collected at 4 time points: before supplementation (day 0), during (day 7), at the end of supplementation (day 28), and after the washout period (day 35) (Figure 1).

Seventy-two healthy Finnish White men and women aged 18–65 years were initially recruited through open calls distributed via university and institutional emailing lists. Prior to enrollment, all participants underwent clinical screening to confirm that they met the inclusion criteria: LDL-cholesterol < 5 mmol/L, TG < 3 mmol/L, and platelet count > 150 × 10^9^/L. Exclusion criteria included fish allergy, pregnancy or breastfeeding, use of prescription pain medication, or ongoing use of lipid-modifying medication or fish oil/vitamin D supplements within 2 weeks prior to study initiation.

Due to COVID-19 restrictions, 27 participants were unable to complete the study. The final study cohort consisted of 38 individuals (11 men and 27 women; see Table 1). Participant disposition is detailed in Supplemental Figure 13. IPE supplementation was generally well tolerated, with several participants reporting fishy breath as the only side effect.

### IPE supplementation

Participants received a daily supply of 6 capsules, each containing 650 mg IPE and 12.5 μg vitamin D_3_, totaling a daily intake of 3.9 g of IPE and 75 μg of vitamin D_3_. They were advised to take 3 capsules in the morning and 3 in the evening with fat-containing meals to facilitate the digestion and hydrolysis of IPE. Capsules were commercially sourced (Midsona Finland Oy). Gas chromatographic analysis of the capsules indicated that 96% of their fatty acid was EPA (Supplemental Table 8).

### Blood sampling and processing

Following an overnight fast, venous blood samples were collected using BD Vacutainers. Plasma samples were immediately placed on ice, while serum samples were incubated at room temperature for 30 minutes. Subsequently, blood components were separated by centrifugation at 4°C: EDTA-plasma at 1,300*g* for 10 minutes and serum at 2,000*g* for 5 minutes. Plasma or serum samples were then immediately aliquoted into 1 mL portions in 1.5 mL Eppendorf polypropylene tubes and stored at –80°C.

### Basic blood count

A basic blood count, including thrombocyte levels, was determined from all the participants prior to their inclusion in the study. EDTA-plasma samples were analyzed at an accredited laboratory (Vita Laboratoriot Oy, Helsinki, Finland) using its automated pipeline. This analysis was performed solely for the baseline samples to assess participant eligibility for the study and was not used in any of the follow up analyses.

### Health checkup

A health checkup was conducted by a practicing physician prior to the study. The assessment included morphological measurements, from which BMI was calculated, as well as blood pressure and blood glucose evaluations. The physician also reviewed the participants’ family history of CVD and other potential disorders, including medications used by the participants. Based on this information, along with a basic blood count, the physician approved or disapproved the participants for the study. One participant was excluded from the study due to low blood platelet levels.

### Chemicals and reagents

Ammonium formate (>99%, UHPLC grade) and ammonium hydroxide (25%, HPLC grade), sucrose (p.a.), sodium chloride (p.a.), calcium chloride (p.a.), magnesium chloride (p.a.), zinc chloride (p.a.), 2-(N-morpholino) ethanesulfonic acid (MES), N-2-hydroxyethylpiperazine-N′-2-ethanesulfonic acid (HEPES), penicillin, streptomycin, and Glutamax, along with lipid standards (SPLASH LIPIDOMIX, ceramide 18:1;O2/12:0) for mass spectrometry, were obtained from Sigma-Aldrich (Merck Life Science, Finland). Fatty acid 13:0 was procured from Larodan Fine Chemicals (Sweden) and deuterium oxide from Cambridge Isotope Laboratories Inc. (USA). The RPMI 1680 medium and fetal bovine serum, along with LC/MS-grade acetonitrile, 2-propanol, methanol, hexane, and chloroform, were purchased from Thermo Fisher Scientific.

### NMR spectroscopy–based metabolomics

NMR spectroscopy–based metabolomics was employed to quantify 250 metabolite and biomarker variables from EDTA-plasma samples at Nightingale Health plc (Helsinki, Finland), following established methods and protocols (74). The platform has been extensively validated (75–77) and remains a widely used and reproducible tool for metabolic profiling in both clinical and research settings. This comprehensive profiling included clinical plasma lipid concentrations, detailed lipoprotein subclass profiles, and various low–molecular weight metabolites, such as glucose, amino acids, and ketone bodies.

### CERT2

The CERT2 is a lipid-based diagnostic test utilized for predicting the risk of myocardial infarction and cardiovascular death (26–29). The CERT2 test was conducted by Zora Biosciences (Espoo, Finland) using EDTA-plasma analyzed by quantitative LC-MS/MS, as detailed previously (27).

### Isolation of lipoproteins by ultracentrifugation

Density-based lipoprotein fractions VLDL+IDL (density 1.019 g/mL), LDL (density 1.063 g/mL), and HDL (density 1.121 g/mL) were isolated from serum using sequential deuterium oxide–based ultracentrifugation, following a protocol adapted from Hallberg et al. (78). The isolation procedures were conducted in a Beckman Optima L-90K ultracentrifuge with a type 50.4Ti rotor at 4°C, operating at 40,000 rpm (172,000*g* average) using Beckman 4 mL open top thick-wall tubes. Initially, 0.5 mL of serum was mixed with 2.14 mL of 140 mM aqueous NaCl (density at room temperature 1.006 g/mL) and 0.36 mL of 140 mM NaCl in deuterium oxide (density at room temperature 1.116 g/mL) to achieve a final density of 1.019 g/mL. After centrifugation for 20 hours, 1 mL of VLDL+IDL was collected from the top layer. To remove residual VLDL-IDL, 1.0 mL of 140 mM NaCl in deuterium oxide was added to the remaining fraction, followed by another 20-hour centrifugation. Subsequently, 1.2 mL of the top layer was removed and discarded.

For the isolation of the LDL fraction, 1.5 mL of 140 mM NaCl in deuterium oxide was added to the remaining bottom fraction to achieve a final density of 1.063 g/mL. Samples were then centrifuged for 72 hours, and 0.5 mL of LDL was collected from the top layer.

To isolate the HDL fraction, 1.3 mL of the remaining homogenous bottom fraction was recovered and mixed with 1.84 mL of 50% sucrose in deuterium oxide (w/w) (density at room temperature 1.315 ± 0.005 g/mL) to achieve a final density of 1.210 g/mL. This mixture was centrifuged for 96 hours, after which 1 mL of HDL was collected from the top layer. The resulting lipoprotein fractions were stored at 4°C prior to assaying.

### Measurement of lipoprotein binding to aortic proteoglycans

To assess the affinity of plasma lipoproteins for human aortic proteoglycans, we employed an ex vivo proteoglycan binding assay, as previously described (13). Briefly, proteoglycans were isolated from the intima-media of human aortas obtained from autopsies, as detailed elsewhere (79), and their glycosaminoglycan (GAG) content was quantified (80). Polystyrene 96-well plates were coated overnight at 4°C with 2.5 μg of proteoglycans (per GAG content) in 100 μL PBS. Nonspecific binding sites were blocked by incubating with 1% BSA in PBS at 37°C for 1 hour. Blank wells without proteoglycan coating served as controls for nonspecific background.

To assess lipoprotein binding, 1 μL of plasma was diluted in 0.1 mL of buffer containing 140 mM NaCl, 2 mM MgCl_2_, 5 mM CaCl_2_, and 10 mM MES (pH 5.5), then added to proteoglycan-coated and control wells, incubating at 37°C for 1 hour. The buffer then was aspirated and discarded, and wells were washed with 10 mM MES/50 mM NaCl (pH 5.5).

The amount of GAG-bound cholesterol in the wells was determined in situ using the Amplex Red cholesterol assay (Molecular Probes). The inter- and intra-assay variance was <2%, determined using pooled plasma obtained from the Finnish Red Cross Blood Service.

### Measurement of LDL aggregation propensity

The propensity of isolated LDL particles to aggregate was assessed using a validated LDL aggregation assay (13, 81). This assay measures the tendency of LDL particles to form aggregates following surface lipid modification by lipolytic enzymes, which are then monitored by dynamic light scattering technology.

Freshly isolated LDL particles were diluted to 0.2 mg/mL in 140 mM NaCl, pH 5.5, and 35 μL of the sample was placed in duplicates on 384-well microplates. Baseline particle size was measured using a DynaPro Plate reader-II with Dynamics software v.7.10.0 (Wyatt Technologies). To induce aggregation, 4 μL of buffer (200 mM MES, 150 mM NaCl, 20 mM CaCl_2_, 20 mM MgCl_2_, 4 mM ZnCl_2_) and 2.5 μL of in-house–produced recombinant sphingomyelinase (0.67 μg/μL in 140 mM NaCl) (81) were added to each well. The wells were sealed with paraffin oil, and particle size was measured every 30 minutes for 7 hours.

Time-versus-size curves were generated, and the inflection point (EC_50_), representing the midpoint of the most rapid aggregation, was calculated by fitting nonlinear regression using a variation of the Hill equation — Y = Bottom + (X^HillSlope^) × (Top-Bottom) / (X^HillSlope^ + EC_50_^HillSlope^) — in GraphPad Prism (v10.1.2.). The aggregation rate was determined as the inverse (1/*x*) of the EC_50_ value for between-subject comparisons. Intra-assay variability was 6.8% (range 5.9%–8.2%), interassay variability was 8.1%, and interoperator variability was 9.6% (11).

### Measurement of cholesterol efflux from macrophages to HDL

The capacity of HDL particles to take up cholesterol from lipid-loaded macrophages was determined using a Cholesterol Efflux Assay kit (MAK192, Sigma-Aldrich) according to manufacturer’s instructions.

Human THP-1 monocytes (ATCC TIB-202, RRID: CVCL 0006) were cultured in RPMI 1640 with 10% fetal bovine serum, 2 mM Glutamax, 100 U/mL penicillin, 100 μg/mL streptomycin, and 25 mM HEPES at 37°C in 5% CO_2_. Cells (~1×10^5^/well) were seeded in a 96-well plate and differentiated into macrophages by incubation with 50 nM PMA for 48 hours, followed by a 24-hour rest in PMA-free medium. Macrophages were then loaded with acetylated LDL containing fluorescently labeled cholesterol for 1 hour, washed, and incubated in equilibration mix for 16 hours.

Serum and isolated HDL were separately tested as cholesterol acceptors for lipid-loaded macrophages. Serum (2 μL) or HDL (20 μg in 100 μL medium) was added to the wells, and after 4 hours, the supernatant was collected for fluorescence measurement. Residual cells were then lysed for 30 minutes, and cell-associated fluorescence was measured. HDL efflux was calculated as the percentage of fluorescence in the supernatant (representing cholesterol transferred to HDL) relative to total fluorescence (supernatant + cell lysates), indicating the ability of HDL or serum to extract cholesterol from macrophages.

### Miscellaneous assays

ELISAs were used to measure the levels of vitamin D (Biohit Oyj, Finland), Lp(a) (Mercodia AB, Sweden), apoC-II, apoC-III (Thermo Fisher Scientific, Finland), and ANGPTL3 (R&D Systems, USA), following the respective manufacturers’ instructions. For vitamin D, EDTA-plasma was assayed for both 25(OH)D_2_ and 25(OH)D_3_. Lp(a), apoC-II, apoC-III, and ANGPTL3 were measured from serum samples.

Total protein concentrations of the lipoprotein fractions (VLDL+IDL, LDL, and HDL) were determined using a Pierce BCA Protein Assay Kit (Thermo Fisher Scientific) with BSA as a standard. NEFAs were determined enzymatically from EDTA-plasma using the Wako NEFA-HR(2) assay kit (Fujifilm).

### Lipoprotein lipid extraction

The lipids of isolated VLDL, LDL, and HDL were extracted as detailed in Supplemental Methods.

### Lipid nomenclature

The abbreviations of lipid classes and species follow previously suggested guidelines (83), except for fatty acids, where the “n-x” nomenclature is applied. Lipid species reported here belong to following lipid classes: fatty acids, phosphatidylcholines (PC), lysophosphatidylcholines (LPC), ether phosphatidylcholines (PC O-), sphingomyelins (SM), steryl esters (SE), cholesteryl esters (CE), ceramides (Cer), and TG. Further information regarding individual lipid species and fatty acid annotations is available in Supplemental Methods.

### LC/MS

LC/MS of lipoprotein lipidomes was performed as detailed in Supplemental Methods.

### GC analyses of plasma total fatty acids

GC analysis of plasma total fatty acids was performed as detailed in Supplemental Methods.

### Procedure for estimating the number of EPA-containing lipid molecules in lipoprotein particles

The process used to estimate the number of lipid molecules containing or not containing EPA in each lipoprotein class (VLDL, LDL, HDL) is described in Supplemental Methods.

### Statistics

#### Normality tests.

The normal distribution of variables was tested using the D’Agostino-Pearson omnibus K2 test using GraphPad Prism v10.1.2.

#### Group differences.

Statistical significances of differences between time points were assessed using paired multiple *t* tests with the Limma method on log_2_ transformed data. Multiple-hypothesis testing was corrected using the false discovery rate. Analyses were performed using the PolySTest tool (82). Prior to group mean comparisons, outliers were identified and removed based on ROUT outlier analysis (Q = 0.01) conducted on GraphPad Prism v10.1.2. All data were combined into a single.csv file, and group differences for all individual variables were calculated in bulk. For certain pairwise comparisons, a paired, 2-tailed Student’s *t* test was used (GraphPad Prism v10.1.2). A *P* value < 0.05 was considered significant.

#### ANOVA.

In specific cases, 1-way ANOVA was used to compare group means. When a significant main effect was detected, post hoc analyses were performed using the 2-stage linear step-up procedure of Benjamini, Krieger, and Yekutieli to control the FDR and identify specific group differences. Analyses were conducted using GraphPad Prism (v10.1.2). A *P* value < 0.05 was considered significant.

#### Correlation analyses.

Spearman’s correlations were used to assess the associations between variables. For datasets with 17 or fewer data pairs, exact *P* values were computed by considering all possible permutations of the data. For larger datasets, approximate *P* values were computed by deriving a *t* ratio from the Spearman’s rank correlation coefficient (Spearman’s rho) and calculating the *P* value from this *t* ratio. This method handles ties and provides accurate results for larger datasets. The analyses were performed using GraphPad Prism (v10.1.2). A *P* value < 0.05 was considered significant.

#### Additional statistical methods.

Time series clustering, PCA, linear mixed modeling, hierarchical clustering, distance calculations, sPLS-DA, UMAPs, and machine learning models are detailed in Supplemental Methods.

### Study approval

The study was approved by the HUS Regional Committee on Medical Research Ethics of the Helsinki University Hospital (Helsinki, Finland) (HUS/2148/2019) and registered at ClinicalTrials.gov (NCT04152291). Written informed consent was obtained from all participants in accordance with the principles of the Declaration of Helsinki.

### Data availability

All data generated in this study are available within the manuscript, its online supplement, and the Supporting Data Values file, which contains data used to generate the figures. In compliance with legislation on anonymity, raw data files are not publicly available, but access to data can be allowed upon reasonable request.

## Author contributions

LÄ, PTK, MJ, M Hermansson, and KÖ conceived the study. LÄ, PTK, and KÖ obtained ethical permissions and organized the clinical trial. LÄ, M Hermansson, MBL, M Holopainen, HR, RK, and RL performed experiments. LÄ, M Hermansson, and KÖ processed data and performed statistical analyses. LÄ, M Hermansson, and KÖ wrote the manuscript, with feedback from all authors.

## Funding support

Jenny and Antti Wihuri Foundation.

Sigrid Jusélius Foundation.

Finnish Cultural Foundation.

Jane and Aatos Erkko Foundation.

Research Council of Finland (332564).

Finnish Foundation for Cardiovascular Research (240087).

Emil Aaltonen Foundation (220244K and 250218KA).

Ida Montin Foundation (20220113 and 20230224).

Novo Nordisk Foundation (NNF10OC0057411).

## Supplementary Material

Supplemental data

ICMJE disclosure forms

Supplemental table 1

Supplemental table 2

Supplemental table 3

Supplemental table 5

Supplemental table 7

Supporting data values

## Figures and Tables

**Table 1:**
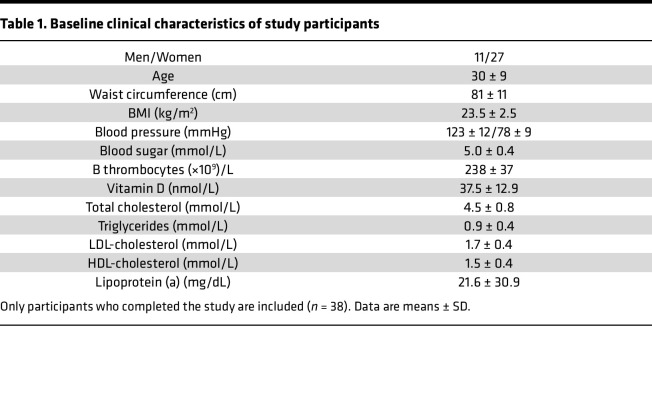
Baseline clinical characteristics of study participants

**Table 2:**
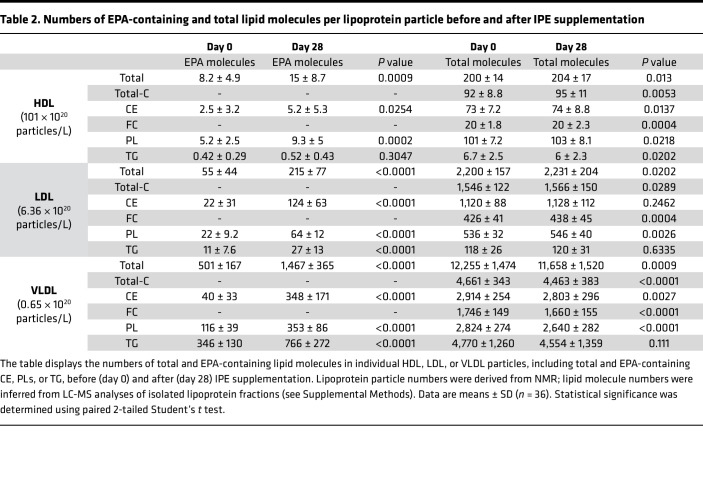
Numbers of EPA-containing and total lipid molecules per lipoprotein particle before and after IPE supplementation

## Version 1 10/08/2025

Electronic publication

## Version 2 10/10/2025

The author affiliation numbers were corrected
