# Supplementary material for: Icosapent ethyl–induced lipoprotein remodeling and its impact on cardiovascular disease risk markers in normolipidemic individuals
Source: JCI Insight. 2025 Oct 8;10(19):e193637. doi: 10.1172/jci.insight.193637 (PMC12513490; doi:10.1172/jci.insight.193637)
Supplement: Supplemental data [file jciinsight-10-193637-s285.pdf]

SUPPLEMENTAL FILE to:

Icosapent ethyl-induced lipoprotein remodelling and its impact on cardiovascular disease risk markers in normolipidemic individuals

Lauri Äikäs[1,2], Petri T. Kovanen[1], Martina B. Lorey[1,2], Reijo Laaksonen[3,4], Minna Holopainen[5], Hanna Ruhanen[5], Reijo Käkelä[5], Matti Jauhiainen[6], Martin Hermansson[1,2], Katariina Öörni[1,2]

[1] Wihuri Research Institute, Helsinki, Finland.
[2] Molecular and Integrative Biosciences Research Programme, Faculty of Biological and Environmental Sciences, University of Helsinki, Helsinki, Finland.
[3] Zora Biosciences Oy, Espoo, Finland. 3
[4] Finnish Cardiovascular Research Center – Tampere, Tampere University, Tampere, Finland.
[5] Helsinki University Lipidomics Unit (HiLIPID), Helsinki Institute of Life Science (HiLIFE), Biocenter Finland, University of Helsinki, Finland.
[6] Minerva Foundation Institute for Medical Research, Helsinki, Finland.

Authorship note: M. Hermansson and KÖ contributed equally to this work.

Address correspondence to: Martin Hermansson or Katariina Öörni, Wihuri Research Institute, Haartmaninkatu 8, 00290 Helsinki, Finland. Phone: 358.50.446.0068; Email: martin.hermansson@helsinki.fi (MH). Email: katariina.oorni@helsinki.fi (KÖ).

Table of Contents

SUPPLEMENTAL TABLES 16

SUPPLEMENTAL METHODS 25

Lipoprotein lipid extraction 25

Lipid nomenclature 25

Liquid chromatography - mass spectrometry analyses of lipoprotein lipidomes 27

Lipid identification and quantification 29

Gas chromatography analyses of plasma total fatty acids 29

Procedure for estimating the number of EPA-containing lipid molecules in lipoprotein particles 30

Statistical methods 33

SUPPLEMENTAL REFERENCES 39

SUPPLEMENTAL FIGURES

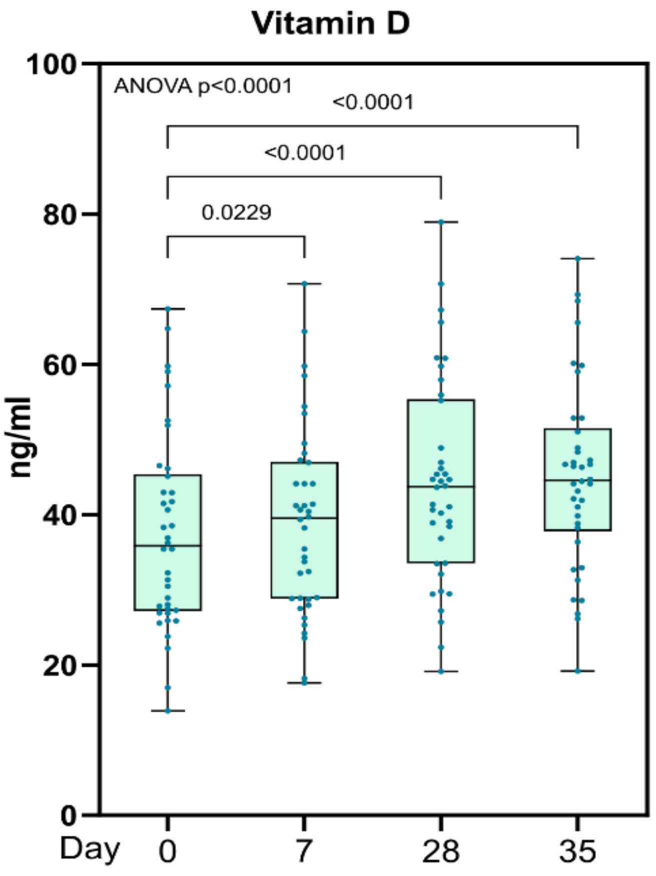

Supplemental Fig. 1. **The impact of the supplement on circulating Vitamin D.** Vitamin D (D3 and D2) were measured from EDTA-plasma using ELISA (*n* = 38). The boxes represent the 25-75th percentile with a median bar, and whiskers represent the range, with all individual data points displayed. Statistical significance of differences between groups was determined using one-way ANOVA with false discovery rate *post hoc* analysis. Please note that the supplement contained Vitamin D (75 µg/Day).

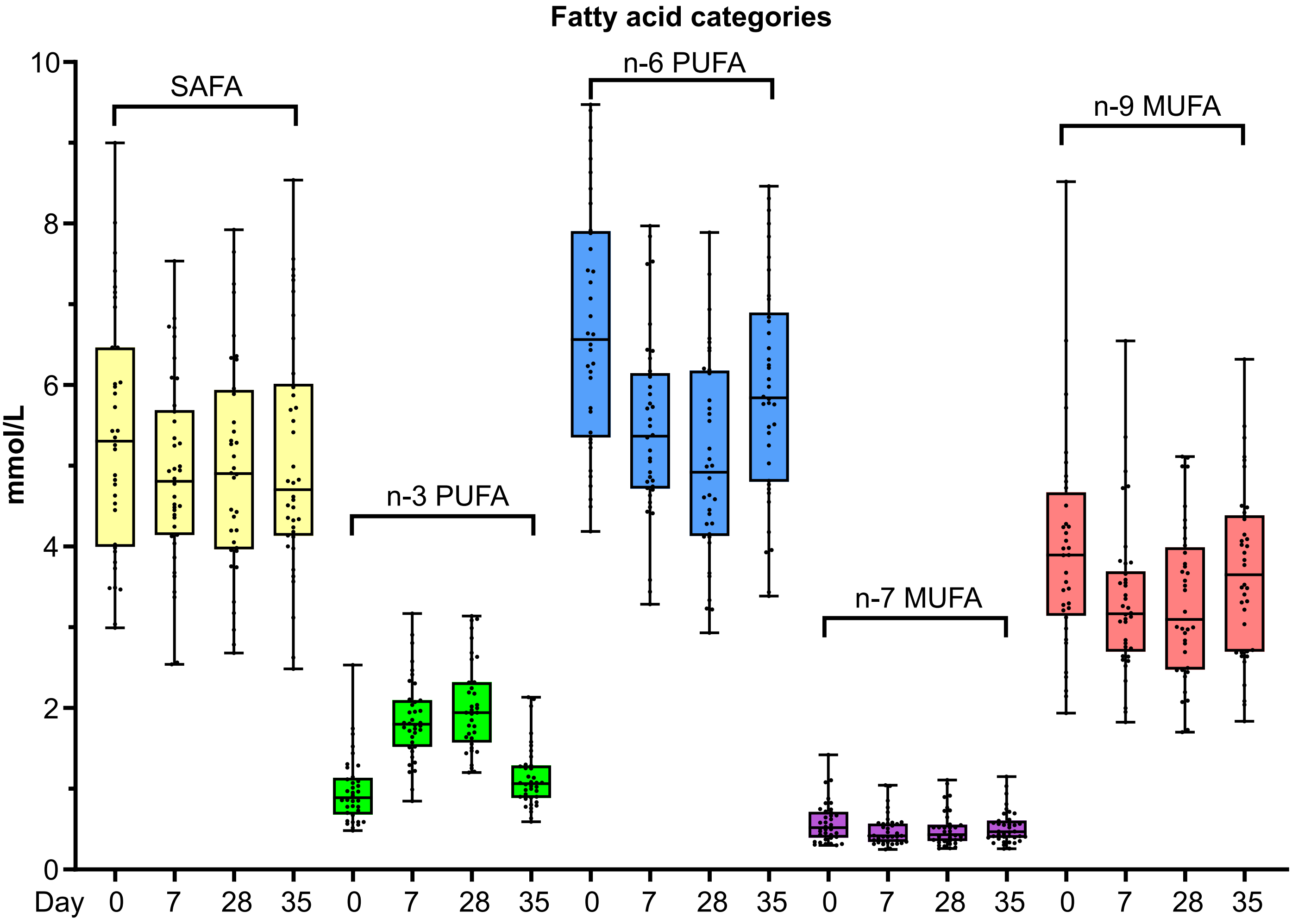

Supplemental Fig. 2. The impact of IPE-supplementation on plasma fatty acid categories. Plasma total fatty acids before (Day 0), during (Day 7), after (Day 28) IPE-supplementation, and after washout period (Day 35), were analysed using gas chromatography (n = 38) and categorized based on their structural features (saturated fatty acids (SAFA), n-3 and n-6 polyunsaturated fatty acids (PUFA), and n-7 and n-9 monounsaturated fatty acids (MUFA). Concentrations of individual fatty acids within each category were summed and are shown as box blots. The boxes represent the 25-75th percentile with a median bar, and whiskers indicate the range, with all individual data points shown. Statistically significant differences for each of the individual fatty acid species are listed in *Supplemental Table 2*.

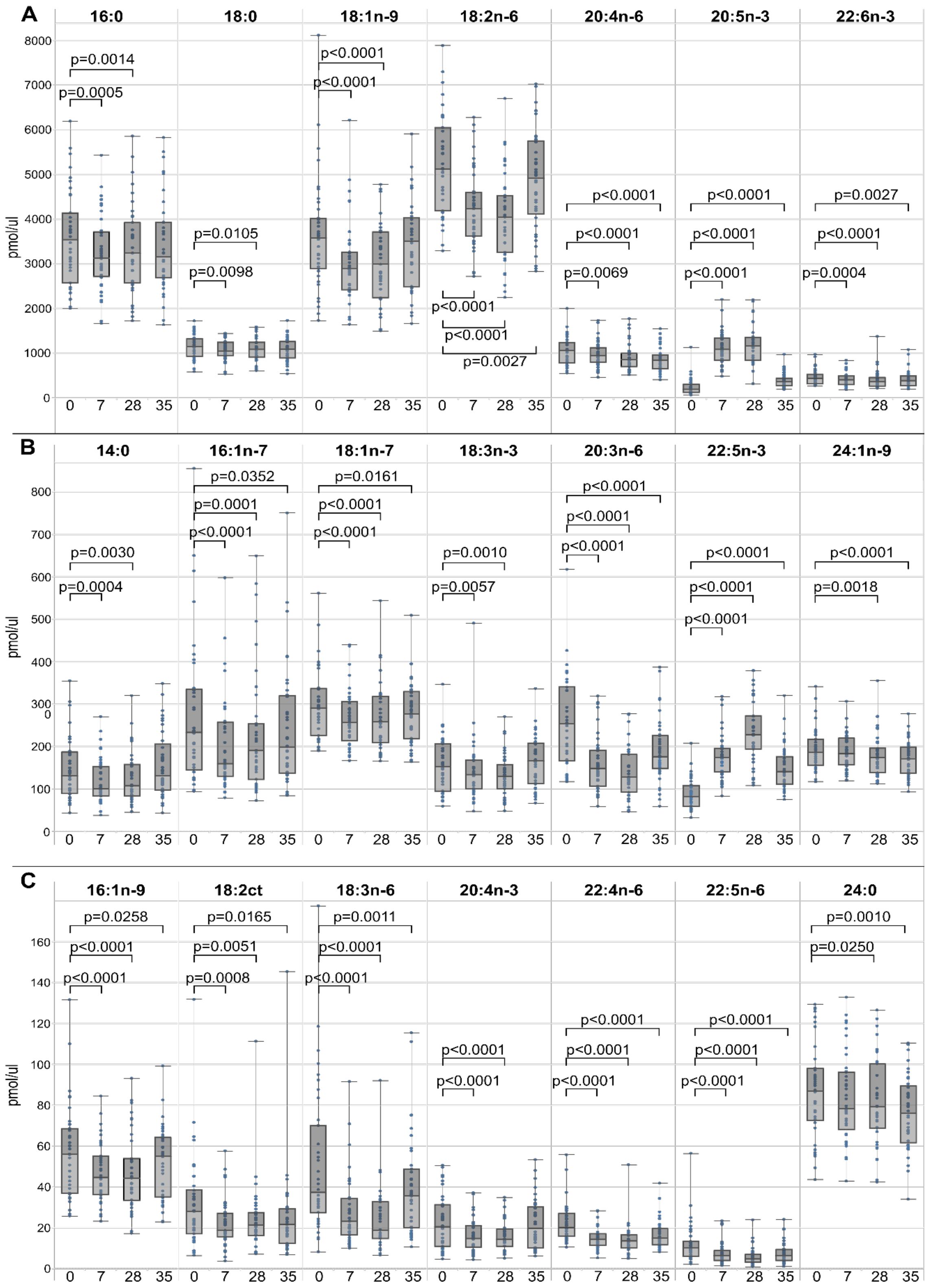

Supplemental Fig. 3. The effect of IPE -supplementation on plasma total fatty acids. Plasma total fatty acids before (Day 0), during (Day 7), after (Day 28) IPE-supplementation, and after washout period (Day 35), were analysed using gas chromatography (n = 36-38). Concentrations of individual fatty acids (>5pmol/µl) are shown as box blots, categorized based on their concentration. The boxes represent the 25-75th percentile with a median bar, and whiskers indicate the range, with all individual data points shown. **A:** Largest concentrations up to 8000 µM, **B:** Medium concentrations up to 800 µM, and **C:** smallest concentrations up to 160 µM. P-values were calculated using the Limma-test with FDR correction for multiple testing, comparing each time point to baseline (Day 0).

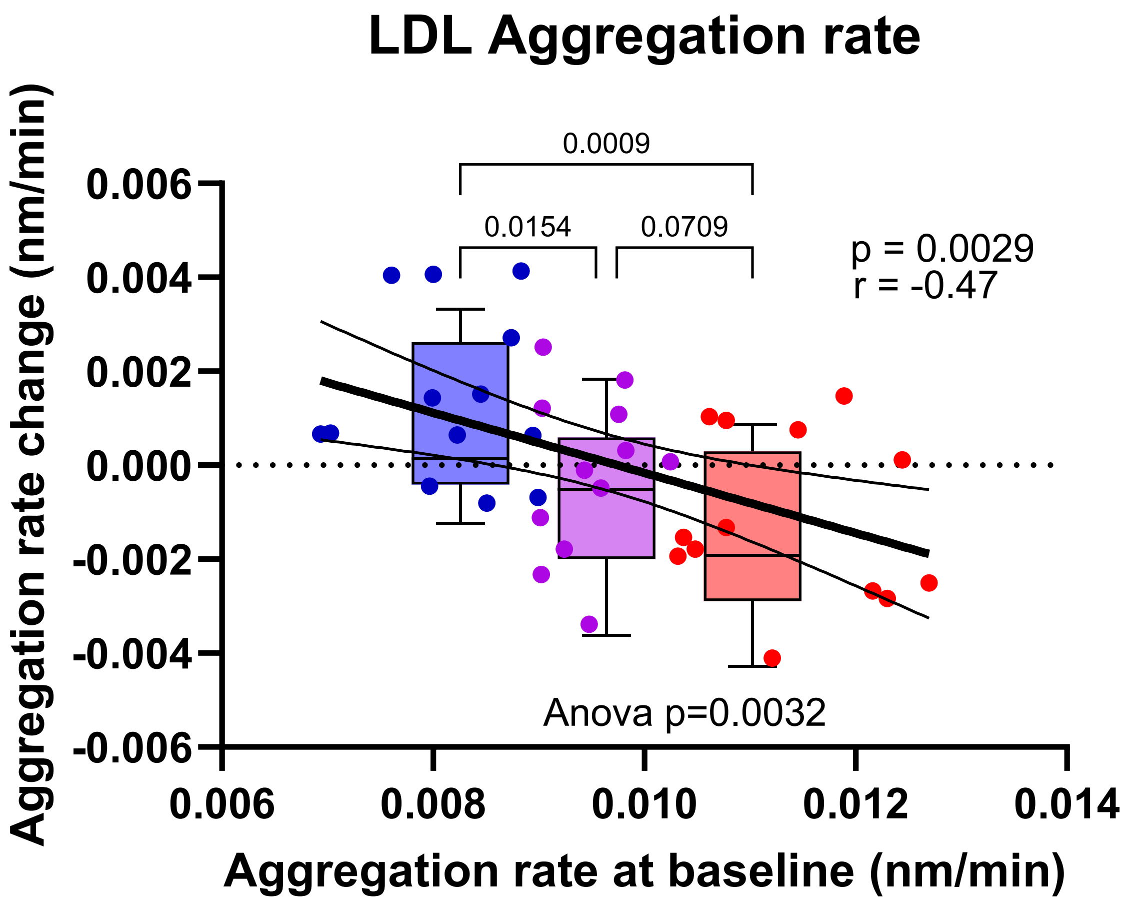

Supplemental Fig. 4. The impact of IPE-supplementation on LDL aggregation rate. LDL aggregation rate was determined as described in Supplemental Methods. The change in LDL aggregation rate after IPE-supplementation (Day 28 minus Day 0; y-axis) was plotted against baseline (Day 0) LDL aggregation rate (x-axis). Aggregation rate indicates the growth of LDL-aggregates per minute (nm/min). Individuals were divided into tertiles based on their baseline aggregation rates; blue = slowest, purple = medium, and red = fastest. The blue tertile, indicating the slowest LDL aggregation, was significantly different from the other tertiles (ANOVA with *post hoc* analysis, adjusted for false discovery rate, n = 38). The change in LDL aggregation rate negatively correlated with the baseline LDL aggregation (Pearson correlation, n = 38).

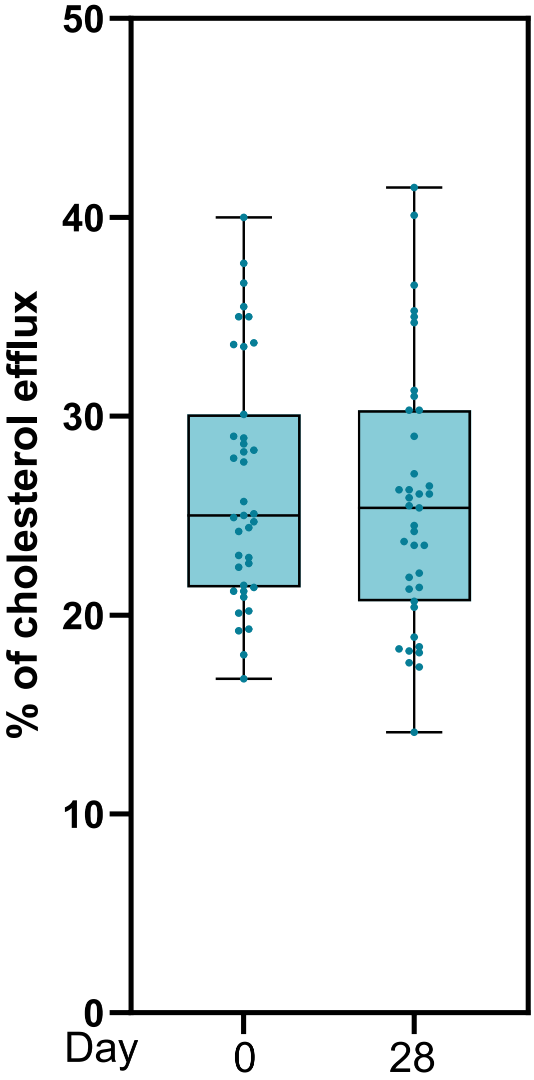

Supplemental Fig. 5. The impact of IPE-supplementation on cholesterol efflux from macrophages to HDL. The capacity of HDL to accept cholesterol from cholesterol-loaded THP-1 macrophages was determined using HDL isolated from plasma drawn before (Day 0) and after IPE-supplementation (Day 28), as detailed in the Supplemental Methods. The boxes represent the 25-75th percentile with a median bar, and whiskers show the range, with all individual data points plotted. There was no statistically significant difference between the groups when assessed by Student's *t*-test (n = 38).

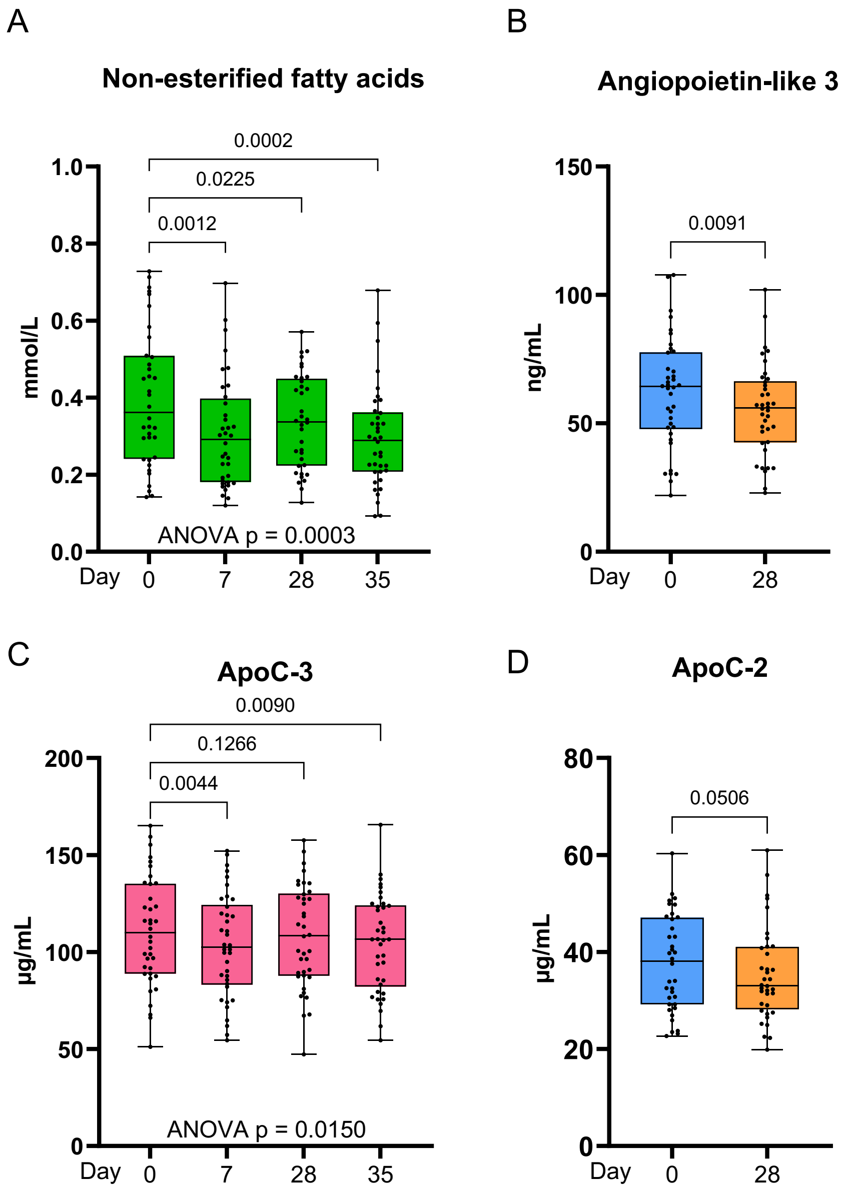

Supplemental Fig. 6. Impact of IPE on plasma non-esterified fatty acid and LPL-cofactor concentrations. For all presented data, the boxes represent the 25-75th percentile with a median bar, and whiskers indicate the range, with all individual data points shown.

A. Plasma non-esterified fatty acids were measured from EDTA-plasma using an enzymatic assay (n = 38) at baseline (Day 0), during (Day 7), after (Day 28) IPE-supplementation, and after washout period (Day 35). The concentrations are presented as box plots. Statistical significance of differences between groups was calculated using ANOVA with *post hoc* analysis, employing FDR correction for multiple testing.
B. Angiopoietin-like 3 was measured from EDTA-plasma using ELISA (n = 38) at baseline (Day 0) and after 28 Days of IPE-supplementation. The statistical significance of the difference between the two time points was calculated using paired Student's *t*-test.
C. ApoC-3 was measured from EDTA-plasma using ELISA (n = 38) before (Day 0), during (Day 7), after (Day 28) IPE-supplementation, and after washout period (Day 35). The statistical significance of the difference between groups was calculated using ANOVA with *post hoc* analysis, employing FDR correction for multiple testing.
D. ApoC-2 was measured from EDTA plasma using ELISA (n = 36) at baseline (Day 0) and after 28 Days of IPE-supplementation. The statistical significance of the difference between the two time points was calculated using paired Student's *t*-test.

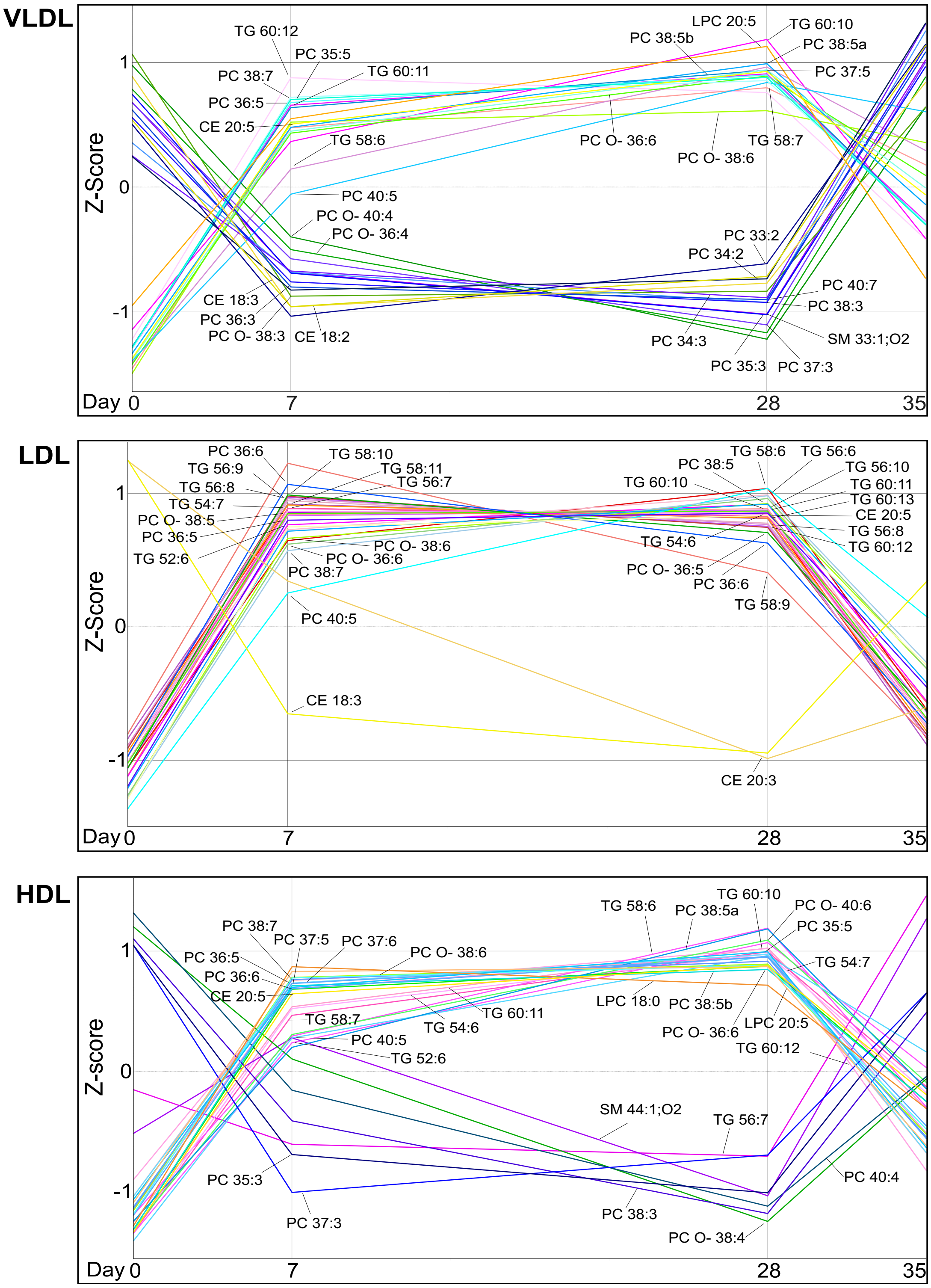

Supplemental Fig. 7. IPE-induced lipid species changes in lipoprotein lipidomes. Plasma was collected from participants at baseline (Day 0), during (Day 7), after (Day 28) IPE-supplementation, and after washout period (Day 35). VLDL, LDL and HDL were isolated using density-based ultracentrifugation, and their lipidomes were analysed using LC-MS. Statistical significance of differences in lipid species abundances between time points were assessed using the Limma-test with FDR correction for multiple testing, comparing each time point to Day 0. Thirty lipid species with lowest *p*-values were chosen for each lipoprotein. The mean abundances of individual lipid species were then z-scaled across all time points. Z-scaling (z-score normalization) standardizes data to have a mean of 0 and a standard deviation of 1, enabling easier comparison across different scales. Data presented from top to bottom: VLDL (n=38) – LDL (n=37) – HDL (n=29).

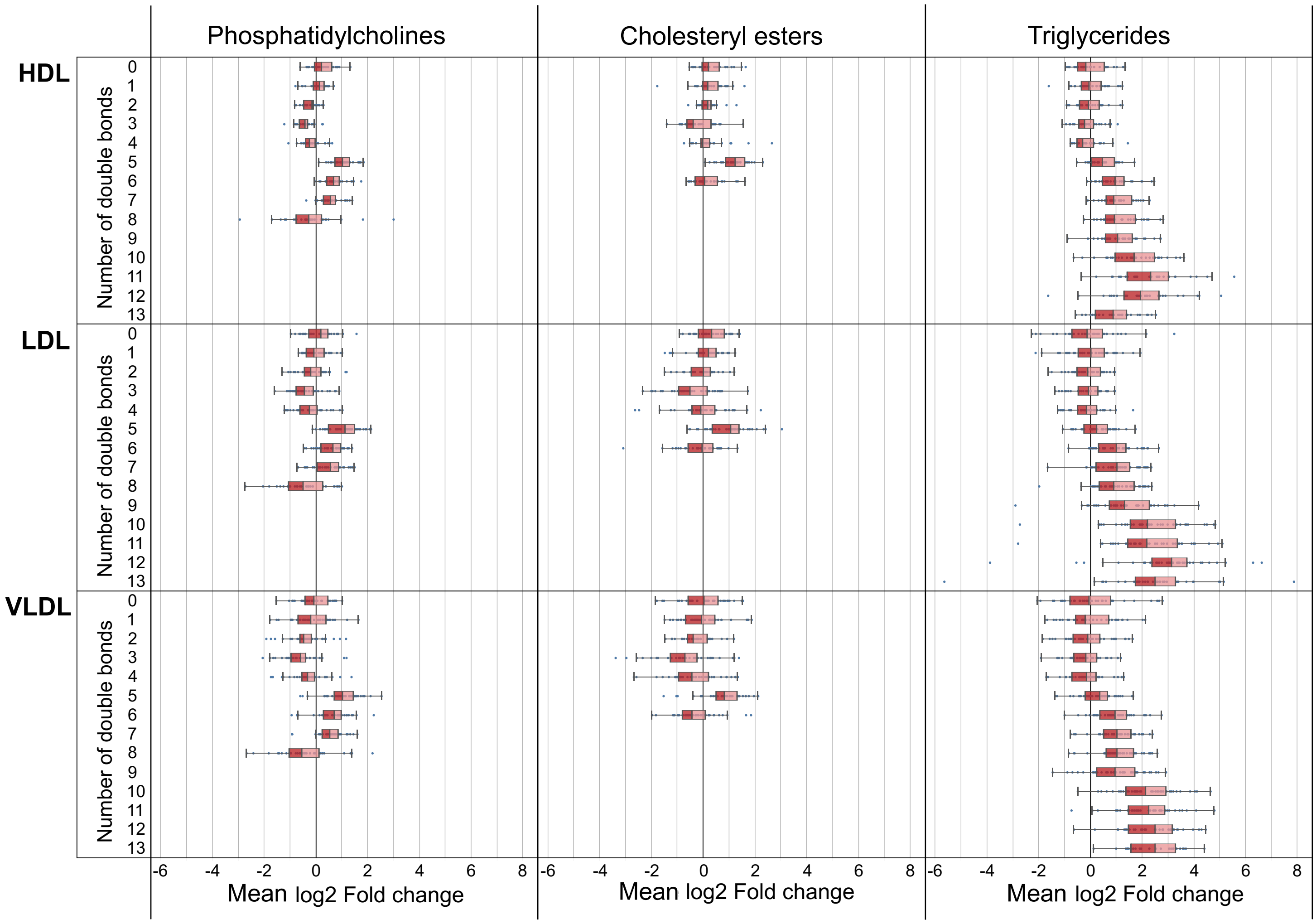

Supplemental Fig. 8. IPE-supplementation increases the unsaturation of lipoprotein lipidomes. Plasma was collected from participants at baseline, and after 28 Days of IPE-supplementation. HDL, LDL and VLDL were isolated using density-based ultracentrifugation, and their lipids were analysed using LC-MS. A log_2 fold change (Day 0 vs. Day 28) was calculated for the abundances (pmol/ µg protein) of individual lipid species in HDL, LDL an VLDL. Lipid species within the PC, CE and TG lipid classes were grouped based on the total number of double bonds in their acyl chains, and a mean log_2 fold change was calculated for each double bond group. The data are presented as box plots, where the boxes represent the 25th to 75th percentiles with a median bar, and whiskers indicate the interquartile range (IQR, k = 1.5), with all individual data points shown.

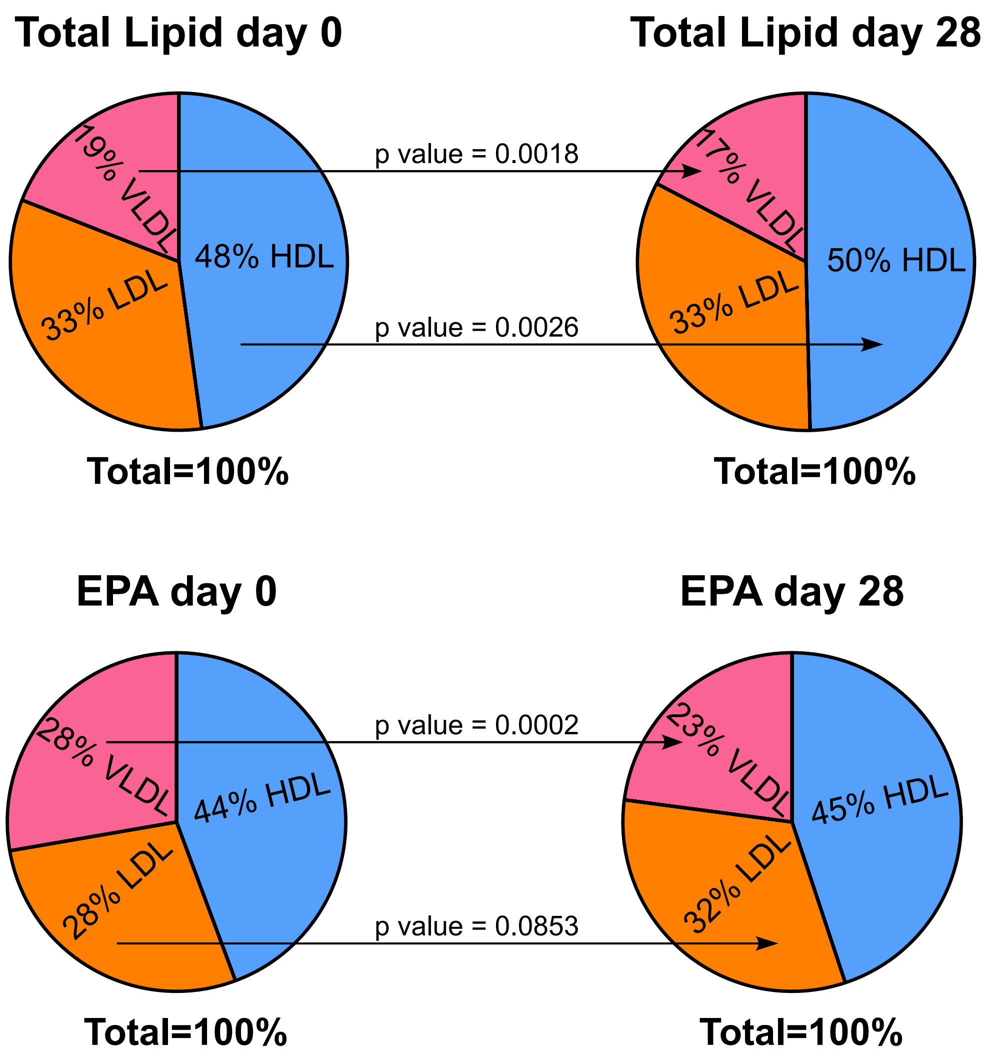

Supplemental Fig. 9. The impact of IPE-supplementation on the distribution of total lipid and EPA among lipoprotein classes.

Top row: The distribution of total lipid associated with VLDL, LDL, and HDL was assessed by NMR spectroscopy at baseline (Day 0) and after 28 Days of IPE-supplementation. Group differences were assessed by Student's *t*-test (n = 29-38), significant differences are indicated.

Bottom row: The distribution of EPA among the main lipoprotein classes was assessed at baseline (Day 0) and after 28 Days of IPE-supplementation, as detailed under Supplemental Methods. Group differences were assessed by Student's *t*-test (n = 29 for HDL, 37 for LDL, and 38 for VLDL), *p*-values are indicated.

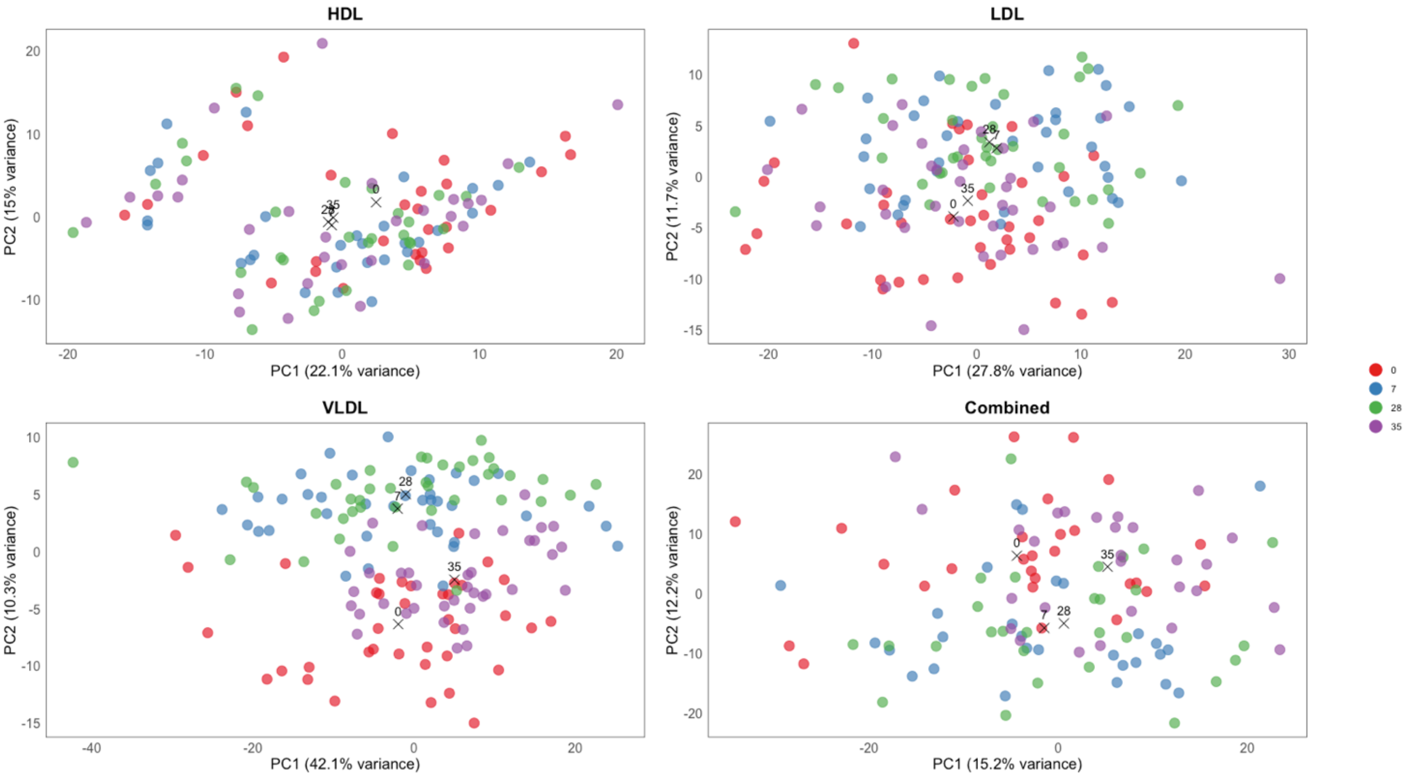

Supplemental Fig. 10. Principal Component Analysis of lipoprotein lipidomes: temporal visualization. Plasma was collected from participants at baseline (Day 0), during (Day 7), after (Day 28) IPE- supplementation, and after the washout period (Day 35). HDL, LDL, and VLDL were isolated using density-based ultracentrifugation, and their lipidomes were analysed using LC-MS (for HDL n = 29, for LDL n = 37, and for VLDL n = 38). PCA analysis was performed on log_2-normalized lipid species abundances for HDL, LDL, and VLDL lipidomes, as well as their aggregate dataset, which includes all lipid species combined for collective analysis. Each point represents a measurement from an individual subject at one of the specified time points, indicated by colour. The axes display the first two principal components (PC1 and PC2), with the percentage of variance explained. Centroids for the distribution of data points were calculated for each time point and are represented as black crosses, labelled accordingly.

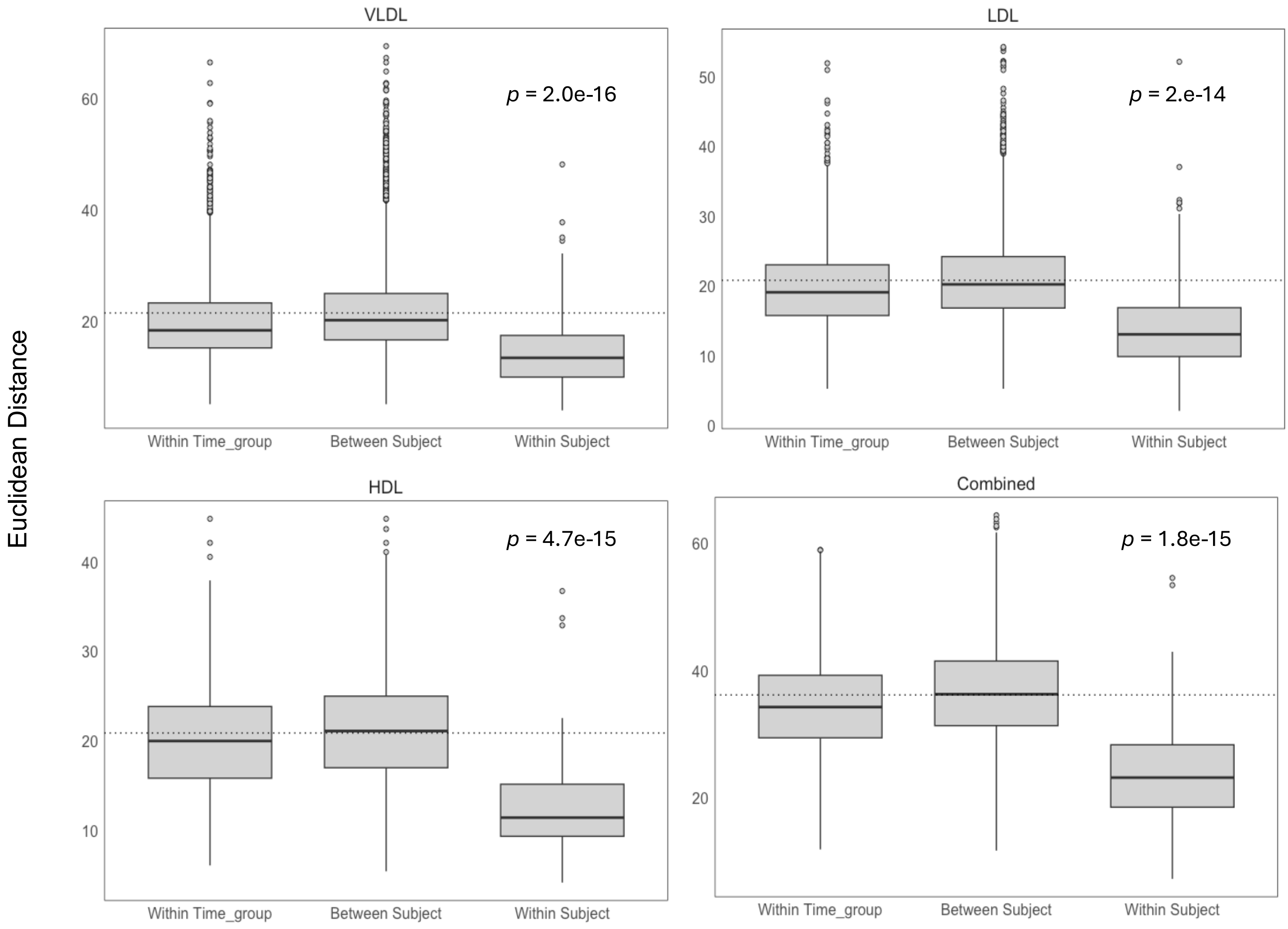

Supplemental Fig. 11. Euclidean distance assessment on the impact of IPE and intra-subject variance on lipoprotein lipidome principal components. PCA was conducted on log_2-normalized lipid species abundances for HDL, LDL, and VLDL lipidomes, as well as their aggregate dataset, which includes all lipid species for collective analysis. Euclidean distances were calculated between individual data points using the first 15 principal components, selected to account for >80% explained variance across all datasets. The dotted line represents the **mean distance** across all data points. The **Within Time Group** represent the distances between observations within each time point, calculated separately and combined to a single box (n = 8612), capturing the IPE-related (Time) variance. **Between Subject Group** measures distances between observations from one subject to those from other subjects (n = 6496-11248), while **Within Subject Group** compares distances among observations from the same subject (n = 174-228). Box plots show the interquartile range (25th to 75th percentiles), with whiskers representing the range up to 2 times IQR. Outliers are shown as individual data points. Differences between groups were assessed pairwise using the Wilcoxon rank-sum test; *p*-values for between subject and within subject comparisons are indicated.

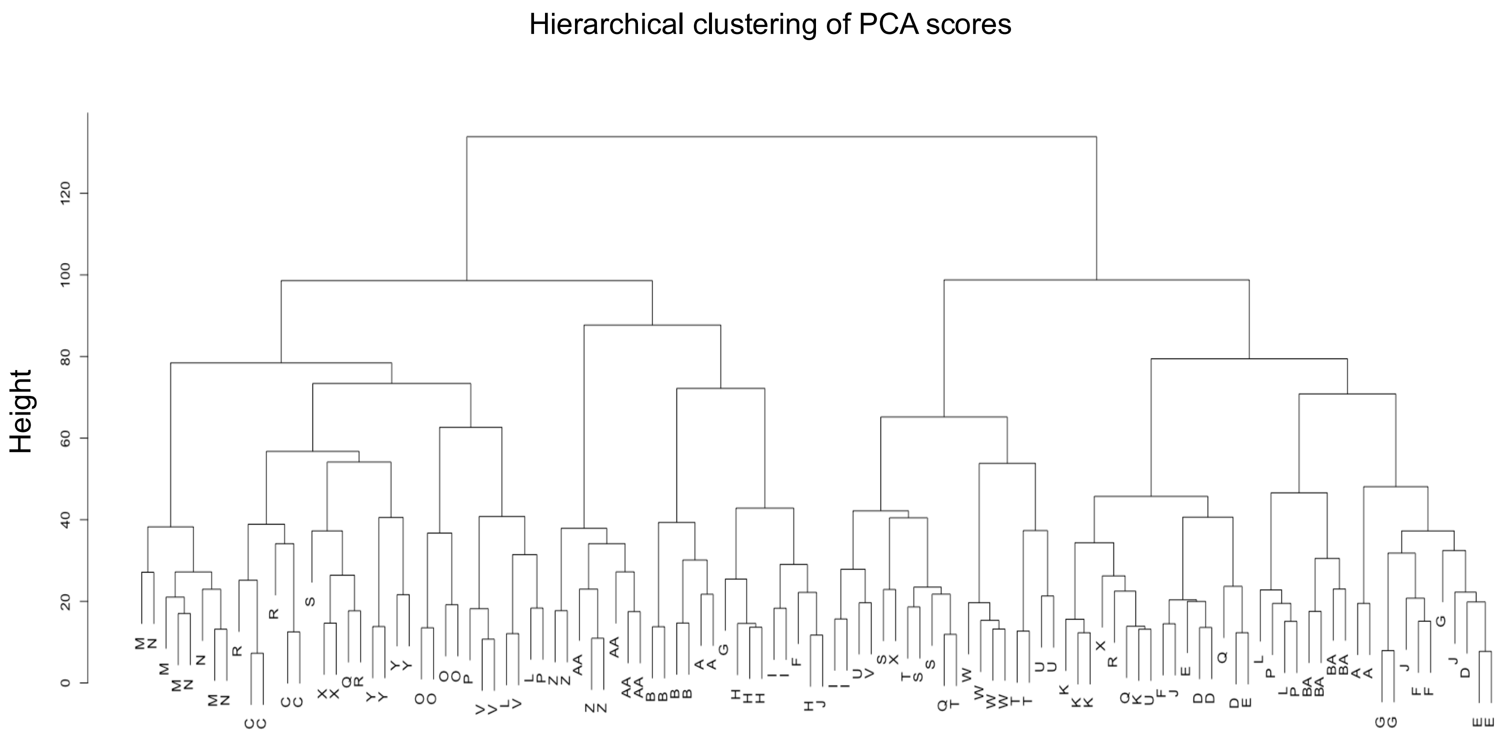

Supplemental Fig. 12. Hierarchical clustering analysis of lipoprotein lipidome principal components. PCA was conducted on the aggregate dataset of log_2-normalized lipid species abundances of VLDL, LDL, and HDL lipidomes, analysed at all four time points (n=116). A Euclidean distance matrix was calculated between all observations based on the first 15 principal components, capturing >80% of the explained variance. Hierarchical clustering, using the Ward.D2 method, was applied to this matrix, and the resulting dendrogram displays the clustering of observations. Each label corresponds to the subject's identifier. Dendrogram height represents the distance between clusters, with lower heights indicating greater similarity. The branching process illustrates how observations are merged based on their similarities, resulting in a hierarchical structure that reveals the relationships among the observations. The cophenetic correlation between the clustering and the original dataset was 0.632.

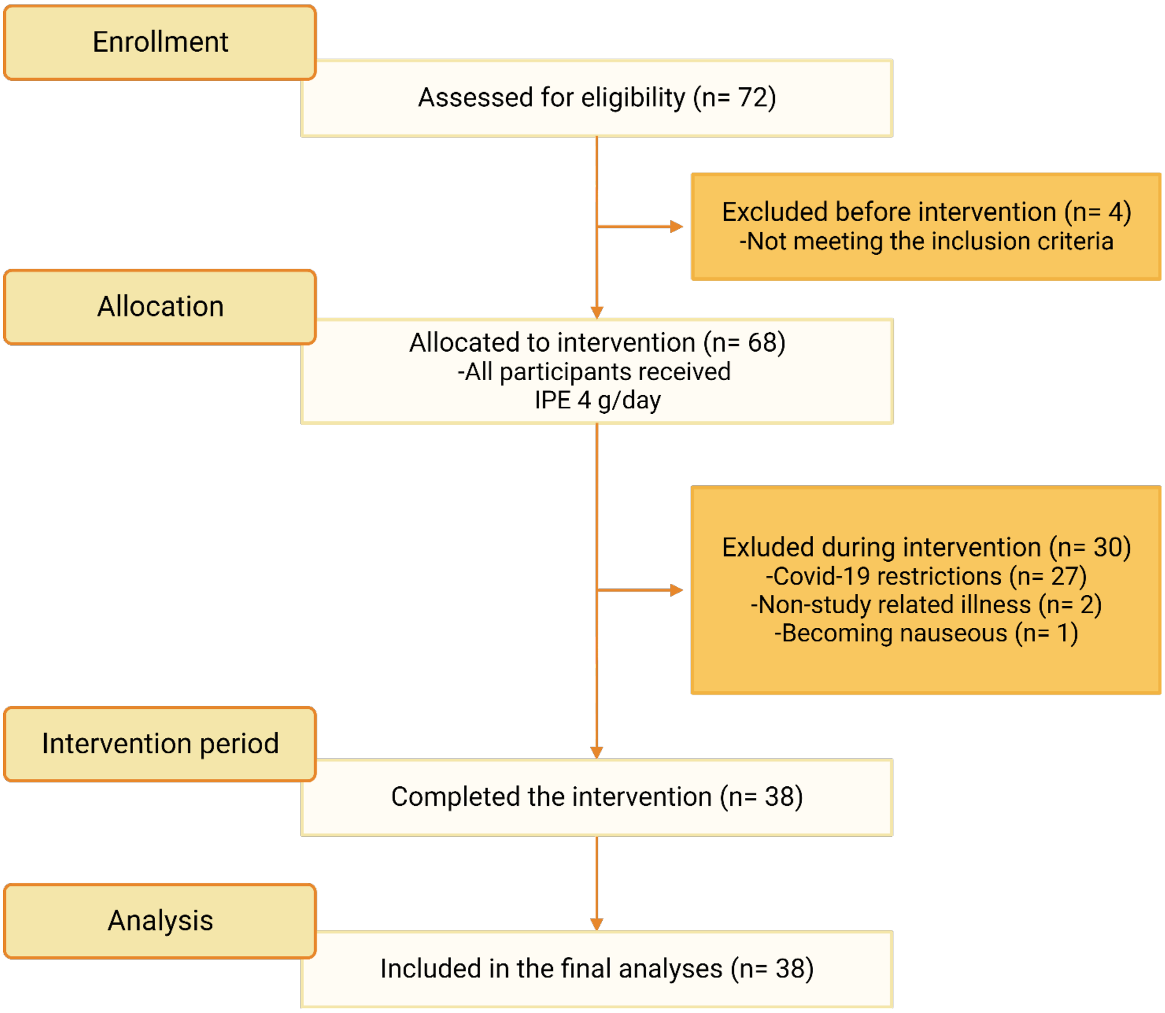

Supplemental Fig. 13. Flow chart of the study cohort.

SUPPLEMENTAL TABLES

Supplemental Table 1

Plasma total fatty acid concentrations before, during and after IPE-supplementation

Hyperlink to Supplemental Table 1.

Plasma total fatty acids before (Day 0), during (Day 7), after (Day 28) IPE-supplementation, and after washout period (Day 35), were analysed using gas chromatography (n = 36-38).

Supplemental Table 1A. A summary of fatty acid concentrations across the different time points. *P*-values were calculated using the Limma-test with FDR correction for multiple testing, comparing each time point to baseline.

Supplemental Table 1B provides a comprehensive output of measurements from each participant.

Supplemental Table 2

Plasma metabolite concentrations before, during and after IPE-supplementation

Hyperlink to Supplemental Table 2.

Plasma metabolite levels before (Day 0), during (Day 7), after (Day 28) IPE-supplementation, and after washout period (Day 35), were analysed using NMR spectroscopy (n = 36-38).

Supplemental Table 2A. A summary of metabolites across the different time points. *P*-values were calculated using ANOVA with FDR correction for multiple testing, comparing each time point to baseline.

Supplemental Table 2B. Provides a comprehensive output of measurements from each participant.

Supplemental Table 3

Plasma lipoprotein (sub)class concentrations before, during and after IPE-supplementation

Hyperlink to Supplemental Table 3.

Plasma lipoprotein levels before (Day 0), during (Day 7), after (Day 28) IPE-supplementation, and after washout period (Day 35), were analysed using NMR spectroscopy (n = 36-38).

Supplemental Table 3A. A summary of lipoprotein (sub)class profiles across the different time points. *P*-values were calculated using ANOVA with FDR correction for multiple testing, comparing each time point to baseline.

Supplemental Table 3B. Provides a comprehensive output of measurements from each participant.

Supplemental Table 4A. Lipid class abundances (pmol/µg protein) in each lipoprotein fraction. Lipid class abundances (pmol/µg protein) of lipoprotein fractions isolated from plasma collected at baseline (Day 0), during IPE-supplementation (Day 7), after IPE-supplementation (Day 28), and following the washout (Day 35). Data are presented as mean ± SD data (n = 38). Differences between groups relative to Day 0 were assessed by ANOVA employing FDR correction for multiple testing. Adjusted *p*-values are shown immediately under abundances.

Lipoprotein	Lipid Class	Day 0	Day 7	Day 28	Day 35
HDL	CER	4 ± 3	4 ± 3	4 ± 4	4 ± 3
			0.8844	0.8844	0.8844
	LPC	22 ± 6	25 ± 8	24 ± 6	22 ± 7
			0.0057	0.0088	0.2191
	PC	873 ± 248	951 ± 202	979 ± 272	950 ± 275
			0.0361	0.0122	0.0361
	PC O-	70 ± 20	79 ± 23	74 ± 25	73 ± 23
			0.0056	0.3154	0.3154
	CE	1370 ± 499	1595 ± 725	1626 ± 726	1625 ± 686
			0.0012	0.0009	0.0037
	SM	146 ± 49	172 ± 67	165 ± 62	160 ± 63
			0.0037	0.0056	0.0322
	TG	112 ± 62	111 ± 44	118 ± 54	152 ± 127
			0.8785	0.5167	0.4052
LDL	CER	2 ± 1	2 ± 1	3 ± 2	2 ± 1
			0.8314	0.7832	0.7832
	LPC	20 ± 7	20 ± 7	20 ± 8	20 ± 7
			0.8227	0.8227	0.8227
	PC	753 ± 205	872 ± 260	847 ± 193	814 ± 260
			0.0128	0.0128	0.0969
	PC O-	65 ± 26	71 ± 26	67 ± 25	66 ± 32
			0.4896	0.8974	0.8974
	CE	3290 ± 1136	3598 ± 1427	3567 ± 1390	3232 ± 1091
			0.3990	0.3990	0.8075
	SM	265 ± 96	299 ± 103	290 ± 103	289 ± 163
			0.1874	0.2322	0.4642
	TG	289 ± 90	320 ± 120	322 ± 115	302 ± 126
			0.1859	0.1859	0.5236
VLDL	CER	11 ± 5	8 ± 5	9 ± 5	12 ± 5
			0.0129	0.0375	0.0129
	LPC	9 ± 3	9 ± 4	9 ± 3	10 ± 3
			0.3743	0.3743	0.3879
	PC	481 ± 181	488 ± 246	514 ± 228	604 ± 177
			0.6061	0.2709	0.0003
	PC O-	31 ± 13	30 ± 13	30 ± 12	35 ± 9
			0.8920	0.8920	0.1059
	CE	789 ± 423	670 ± 468	708 ± 416	871 ± 362
			0.1475	0.1475	0.1824
	SM	89 ± 39	77 ± 39	81 ± 42	94 ± 35
			0.1571	0.1879	0.2478
	TG	1131 ± 485	1170 ± 654	1279 ± 706	1535 ± 589
			0.4836	0.0937	0.0001

Supplemental Table 4B. Lipid class composition (mol%) of each lipoprotein fraction. Lipid class compositions (mol%) of lipoprotein fractions isolated from plasma collected at baseline (Day 0), during IPE-supplementation (Day 7), after IPE-supplementation (Day 28), and following the washout (Day 35). Data are presented as mean ± SD data (n = 38). Differences between groups relative to Day 0 were estimated using ANOVA employing FDR multiple testing correction. Adjusted *p*-values are shown immediately under mol% values.

Lipoprotein	Lipid Class	Day 0	Day 7	Day 28	Day 35
HDL	CER	0.2 ± 0	0.1 ± 0<br>0.2170	0.1 ± 0<br>0.2170	0.1±0<br>0.2170
	LPC	0.8 ± 0	0.9 ± 0<br>0.3786	0.8 ± 0<br>0.2332	0.7 ± 0<br>0.0043
	PC	34.6±9	34.4±10<br>0.7839	34.4±10<br>0.7839	33.4±9<br>0.4473
	PC O-	2.8±1	2.9±1<br>0.1349	2.6±1<br>0.0115	2.6±1<br>0.0039
	CE	51.7±8	52.2±10<br>0.5471	52.5±10<br>0.4950	52.7±9<br>0.4950
	SM	5.7±1	5.8±1<br>0.6971	5.6±2<br>0.6971	5.4±1<br>0.6971
	TG	4.2±1	3.8±1<br>0.2364	3.9±1<br>0.2364	5±3<br>0.2364
LDL	CER	0±0	0±0<br>0.181	0±0<br>0.8258	0±0<br>0.3994
	LPC	0.4±0	0.4±0<br>0.2683	0.4±0<br>0.2683	0.4±0<br>0.8237
	PC	16.5±2	17.2±2<br>0.2338	17.2±3<br>0.2338	17.7±4<br>0.2338
	PC O-	1.4±0	1.4±0<br>0.9943	1.3±0<br>0.9266	1.4±0<br>0.9943
	CE	69.6±4	68.7±3<br>0.3227	68.6±5<br>0.3227	67.9±5<br>0.3227
	SM	5.7±1	5.9±1<br>0.6074	5.8±1<br>0.7714	6±2<br>0.6074
	TG	6.4±2	6.3±2<br>0.7983	6.5±2<br>0.7983	6.5±2<br>0.7983
VLDL	CER	0.4±0	0.4±0<br>0.0030	0.4±0<br>0.0122	0.4±0<br>0.0235
	LPC	0.4±0	0.4±0<br>0.3485	0.4±0<br>0.3485	0.3±0<br>0.0036
	PC	19.3±2	20.6±3<br><0.0001	20.3±3<br><0.0001	19.3±3<br>0.3161
	PC O-	1.3±0	1.3±0<br>0.0125	1.2±0<br>0.0464	1.1±0<br><0.0001
	CE	30±8	25.5±8<br><0.0001	26.1±8<br><0.0001	27.7±8<br>0.0169
	SM	3.5±1	3.2±1<br>0.0009	3.1±1<br><0.0001	3±1<br><0.0001
	TG	45±7	48.6±7<br>0.0002	48.5±7<br>0.0002	48.1±8<br>0.0016

Supplemental Table 5

VLDL, LDL and HDL lipidomes before, during and after IPE-supplementation

Hyperlink to Supplemental Table 5.

Isolated lipoproteins (VLDL, LDL, and HDL) lipidomes before (Day 0), during (Day 7), after (Day 28) IPE-supplementation, and after washout period (Day 35), were analysed using LC-MS. N = 29 for HDL, 37 for LDL, and 38 for VLDL.

Supplemental Table 5A. A summary of VLDL, LDL and HDL lipid profiles across the different time points. *P*-values were calculated using LIMMA with false discovery rate correction for multiple testing, comparing all time points to baseline.

Supplemental Table 5B. Provides a comprehensive output of measurements from each participant.

Supplemental Table 6. Linear Mixed Modelling of lipoprotein lipidome principal components. PCA was performed separately on log_2-normalized lipid species abundances for HDL, LDL, VLDL lipidomes, and a combined dataset incorporating all lipid species. The **Dataset** is indicated on the left. **Total variance** explained by the first 10 principal components (PC) for each dataset are reported. Linear mixed-effects modelling assessed the influence of the fixed effect (Time, reflecting the impact of IPE-supplement) and random effect (Subject, accounting for repeated measures). **Fixed effect variance** indicates the proportion of total variance attributed to time-based differences, while **Random effect variance** accounts for subject-specific variability. **Marginal R^2** represents the variance explained by the fixed effect, and **Conditional R^2** reflects the total variance explained by both fixed and random effects. Marginal and conditional R^2 values are calculated based on the variance components of the model, following the method by Nakagawa & Schielzeth (1), and represent the variance explained by the fixed effects and the combined fixed and random effects, respectively. The **Fixed effect estimate** indicates the average shift in PC values between time points, with *p*-values representing its significance. The *p*-values are calculated based on *t*-values, which assess the significance of the fixed effect relative to its standard error.

Dataset	PC	Total variance	Fixed effect variance	Random effect variance	Marginal R^2	Conditional R^2	Fixed effect estimate	*p*-value
**HDL**	PC1	68.8	0.8	52.9	0.01	0.78	-1.81	1.5E-02
	PC2	47.0	0.7	37.0	0.01	0.80	-1.63	5.3E-03
	PC3	26.7	16.8	3.8	0.63	0.77	8.19	4.1E-34
	PC4	21.1	0.4	18.2	0.02	0.88	-1.31	2.6E-05
	PC5	15.9	0.2	11.6	0.01	0.74	-0.84	3.0E-02
	PC6	12.7	0.5	5.4	0.04	0.46	-1.35	6.8E-03
	PC7	7.3	0.0005	4.3	0.0001	0.58	-0.04	8.9E-01
	PC8	6.8	0.007	5.8	0.001	0.85	-0.17	3.7E-01
	PC9	5.9	0.1	4.2	0.02	0.74	0.70	3.4E-03
	PC10	5.3	0.03	2.6	0.01	0.48	0.33	2.9E-01
**LDL**	PC1	86.8	2.4	35.4	0.03	0.44	3.12	8.0E-03
	PC2	36.6	9.6	17.3	0.26	0.73	6.22	1.6E-23
	PC3	27.7	5.1	15.4	0.18	0.74	4.54	1.0E-18
	PC4	20.2	2.8	13.1	0.14	0.79	3.39	7.6E-18
	PC5	13.9	0.003	11.1	0.0002	0.79	0.11	6.9E-01
	PC6	9.4	0.0	7.2	0.01	0.78	0.44	7.0E-02
	PC7	7.5	0.2	4.3	0.03	0.61	-0.97	7.6E-04
	PC8	6.2	0.1	2.3	0.02	0.38	0.63	5.4E-02
	PC9	5.9	0.1	3.8	0.01	0.67	0.54	2.1E-02
	PC10	5.0	0.01	3.0	0.002	0.60	-0.20	3.9E-01
**VLDL**	PC1	132.7	2.3	60.9	0.02	0.48	-3.06	2.6E-02
	PC2	32.5	19.4	5.2	0.60	0.76	8.79	8.4E-42
	PC3	22.6	0.02	18.2	0.001	0.81	0.28	4.2E-01
	PC4	15.5	1.0	13.2	0.07	0.92	2.03	5.0E-21
	PC5	13.8	0.5	11.5	0.04	0.87	-1.42	1.0E-09
	PC6	9.1	0.3	6.4	0.04	0.74	-1.18	6.5E-06
	PC7	7.4	0.02	6.3	0.003	0.86	0.30	7.3E-02
	PC8	6.3	0.01	3.4	0.002	0.53	-0.20	4.7E-01
	PC9	4.4	0.04	3.1	0.01	0.72	0.39	3.4E-02
	PC10	3.7	0.004	2.2	0.001	0.60	0.13	5.1E-01
**Combined**	PC1	142.3	0.2	60.2	0.001	0.42	-0.86	6.2E-01
	PC2	114.6	28.8	51.5	0.25	0.70	-10.76	3.5E-16
	PC3	77.7	21.7	39.3	0.28	0.78	9.34	2.0E-21
	PC4	73.1	6.5	58.7	0.09	0.89	-5.15	3.5E-16
	PC5	63.9	2.1	41.4	0.03	0.68	-2.89	1.1E-03
	PC6	45.4	2.1	37.3	0.05	0.87	-2.92	8.5E-09
	PC7	43.0	0.03	29.0	0.001	0.67	0.34	6.4E-01
	PC8	24.9	0.3	15.0	0.01	0.62	1.14	5.7E-02
	PC9	20.7	0.1	17.7	0.01	0.86	0.67	4.1E-02
	PC10	19.7	0.05	12.6	0.002	0.64	0.44	3.9E-01

Supplemental Table 7

Spearman's correlation analysis of clinical biomarkers, plasma metabolites, and lipoprotein lipidomes with lipoprotein proteoglycan-binding affinity and LDL aggregation rate

Hyperlink to Supplemental Table 7.

The affinity of plasma lipoproteins for aortic proteoglycans, or LDL aggregation propensity were determined as described in Supplemental Methods. A Spearman's correlation analysis of these parameters was then conducted against all other parameters measured in this study, as detailed under Supplemental Methods.

Supplemental Table 8. Fatty acid composition of the IPE-supplement. Three randomly selected IPE-capsules were analysed using gas chromatography. The identified fatty acids are presented in the table as mol% of total. The relative amounts of total saturated (SAFA), monounsaturated (MUFA), and polyunsaturated (PUFA) fatty acids, as well as n-6 and n-3 PUFAs, are also included.

Mol% of total	Capsule 1	Capsule 2	Capsule 3
18:0	0.22	0.24	0.22
18:1n-9	0.28	0.31	0.29
18:1n-7	0.12	0.12	0.11
18:4n-3	0.27	0.26	0.25
20:1n-9	0.34	0.33	0.33
20:4n-6	1.19	1.20	1.20
20:4n-3	1.51	1.52	1.50
20:5n-3	96.08	96.03	96.10
Total SAFA	0.22	0.24	0.22
Total MUFA	0.73	0.75	0.74
Total PUFA	99.04	99.01	99.05
Total n-6 PUFA	1.19	1.20	1.20
Total n-3 PUFA	97.85	97.81	97.85

SUPPLEMENTAL METHODS

Lipoprotein lipid extraction

Lipoprotein subclasses (VLDL, LDL or HDL) were isolated from blood plasma by density gradient centrifugation (*cf.* isolation of lipoproteins) and the protein content of isolated fractions was assessed by a BCA-assay. The extractions were carried out in 1.5 ml LoBind microcentrifuge tubes (Eppendorf, Germany). On total protein basis, 5-10 µg of lipoprotein was diluted with 0.2 M ammonium formate to a total volume of 0.2 ml, spiked with internal lipid standards and then mixed with 0.75 ml Chloroform-Methanol 1:2 (CM 1:2, v/v). The samples were placed in a ThermoMixer (Eppendorf, Germany) and mixed for 15 min at 1400 rpm at RT. Then, 0.25 ml 0.2 M ammonium formate and 0.25 ml chloroform were added, and the samples were mixed further for 15 min at 1400 rpm at RT. The phases were then separated by centrifugation for 5 min at 5000 x g at +4 °C. The lower phase was transferred to a new tube and the remaining aqueous phase was re-extracted with 0.5 ml chloroform by mixing for 15 min at 1400 rpm at RT followed by centrifugation for 5 min at 5000 x g at +4 °C. The lower phase was recovered and pooled with the previous lower phase, evaporated by vacuum evaporator, reconstituted in 0.25 ml of CM 1:2, transferred to 1.5 ml borosilicate glass sample vials, capped and stored at – 20 °C.

Lipid nomenclature

The abbreviations of lipid classes and species follow previously suggested guidelines (2), except for fatty acids, where the "n-x" nomenclature is applied. Lipid species reported here belong to following lipid classes: fatty acids (FA), phosphatidylcholines (PC), lysophosphatidylcholines (LPC), ether phosphatidylcholines (PC O-), sphingomyelins (SM), steryl esters (SE), cholesteryl esters (CE), ceramides (Cer) and triglycerides (TG).

At the lipid species level, glycerolipids and glycerophospholipids are denoted as: <lipid class> <total number of carbons in hydrocarbon (acyl/alkyl) moieties>:<total number of double bonds in hydrocarbon (acyl/alkyl) moieties>. For example, "PC 38:5" denotes a PC with 38 carbons and 5 double bonds spread across both individual fatty acid chains. For ether glycerophospholipids, ether-bound hydrocarbon chains are preceded with an "O" indicating either 1-O-alkyl ether or 1-O-alkenyl ether linkage (e.g. PC O-38:5). Sphingolipid species are denoted as <lipid class> <total number of carbons in the long-chain base and acyl moiety>:<total number of double bonds in the long-chain base and fatty acyl moiety>;O<total number of OH groups in the long-chain base and acyl moiety> (e.g. SM 36:1;O2). SEs are denoted as <lipid class> <total number of C in the sterol backbone and acyl moiety>:<total number of double bonds in the sterol backbone and acyl moiety> (e.g. SE 47:6).

When MS/MS fragmentation (see below) supports annotation at the molecular lipid species level, they are denoted as: <lipid class> <total number of carbons in the in the long-chain base>:<total number of double bonds in the long-chain base>;O<total number of OH groups in the long chain base>/<total number of carbons in the acyl moiety>:<total number of double bonds in the acyl moiety> (e.g. Cer 18:1;O2/24:0). Since cholesterol (ST 27:1) is by far the most abundant sterol

carried in lipoproteins, and when annotation is supported by fragmentation, SE species are annotated as CE, where the CE indicates a ST 27:1 backbone, followed by <total acyl carbons>:<total double bonds in the acyl moiety> (e.g. CE 20:5).

The annotation of FAs follows the n-x nomenclature, i.e., <total carbon number>:<total number of double bonds>n-<position of the first double bond calculated from the methyl end>, e.g., 20:5n-3 for eicosapentaenoic acid. The n- is omitted for saturated fatty acids that do not contain double bonds.

Liquid chromatography - mass spectrometry analyses of lipoprotein lipidomes

a) Liquid chromatography

The chromatographic equipment and conditions are specified in table the below.

**LC-MS instrumentation and settings**	
**Instrumentation**	
Liquid chromatography	ACQUITY UPLC system (Waters, UK)
Columns	ACQUITY UPLC BEH C18 (1 × 100 mm, 1.7 µm),<br>ACQUITY UPLC BEH C18 VanGuard (2.1 × 5 mm, 1.7 µm)
Mass spectrometry	Quattro Premier TQ (Waters, UK)
Source	Electrospray ionisation (ESI)
Coordinating LC-MS system	MassLynx V4.1 (Waters, UK)
**Liquid chromatography**	
Needle wash (strong)	1:1 Acetonitrile:2-propanol
Needle wash (weak)	1:1 Acetonitrile:H2O
Mobile phase A	600:400:10 H_2O:Acetonitrile:NH_4OH (v/v) with 10 mM ammonium formate
Mobile phase B	900:100:10 2-propanol:Acetonitrile:NH_4OH (v/v) with 10 mM ammonium formate
Injection	5 µL, 1:2 CHCl3:MeOH, partial-loop injection
Inlet gradient elution	0-10 min (40-70% B), 10-14 min (70-100% B), 14-16 min (100% B), 16-19min (40% B), 19-23min (40% B)
Flow rate	0.13 mL min^{-1}
Column temperature	50 °C
**Mass spectrometry**	
Nebulizing gas	Nitrogen
Desolvation gas	N_2 (600 L/hr; 300°C)
Collision gas	Argon (99.999%)
Capillary voltage	mode-dependent
Analysis mode	Positive
Acquisition mode	MS+, PIS184, PIS369, Multiple reaction monitoring (MRM)
Acquisition rate	mode-dependent
Mass resolution	Unit
**Data processing**	
Processing software	QuanLynx V4.1 (Waters, UK); Microsoft excel; Orange3; Tableau

Briefly, an aliquot of the lipid extract in chloroform:methanol 1:2 was transferred into 0.3 ml inserted polypropylene sample vials (Waters, UK) and placed in the ACQUITY autosampler, which

was operated at 7 °C. The injection volume was 5 µl, using a partial loop injection. Chromatographic separations were performed on an ACQUITY Ultra-Performance Liquid Chromatography system (Waters Corp, Milford, MA) using a 1 mm × 100 mm ACQUITY BEH 1.8 µm C18 analytical column (Waters Corp, Milford, MA) fitted with an ACQUITY in-line 0.2 µm pre-filter.

The mobile phase was composed of solvents A (water:acetonitrile:ammonium hydroxide 60:40:1 with 10 mM ammonium formate) and B (2-propanol:acetonitrile:ammonium hydroxide 90:10:1 with 10 mM 10 mM ammonium formate). The column was maintained at 50 °C and eluted at a flow rate of 0.13 ml/min with a gradient starting from 40% B, linearly increasing to 70% B over 10 minutes, then to 100% B over 4 minutes. After holding at 100% B for two minutes, it returned to 40% in 3 minutes and held for 3 minutes before next injection.

b) Mass spectrometry

The MS equipment and conditions are specified in tables "LC MS instrumentation and settings" and "Instrument parameters" .

	**Acquisition mode**			
**Instrument Parameters**	**MS+**	**PIS184**	**PIS369**	**MRM(264)**
Ionization Mode	ES+	ES+	ES+	ES+
Calibration	Dynamic 1	Dynamic 1	Dynamic 1	Static 2
Capillary (kV)	3.6	3.6	3.4	3
Cone (V)	40	40	40	20
Extractor (V)	3	3	3	3
RF Lens (V)	0.3	0.3	0.3	0.3
Source Temperature (°C)	100	100	100	100
Desolvation Temperature (°C)	300	300	120	300
Cone Gas Flow (L/Hr)	50	50	10	35
Desolvation Gas Flow (L/Hr)	600	600	600	600
LM 1 Resolution	15	15	15	14
HM 1 Resolution	15	15	15	14
Ion Energy 1	1	1	3	3
Entrance	50	1	-1	-1
Collision	2	30	15	30
Exit	50	2	1	1
LM 2 Resolution	14.5	14.5	14.5	14
HM 2 Resolution	14.5	14.5	14.5	14
Ion Energy 2	1	1	1	1
Multiplier (V)	650	650	650	650
Syringe Pump Flow (uL/min)	0	0	0	0
Pressure (m bar)	< 1E-4	2.89E-03	3.05E-03	3.03E-03
Collision Gas Flow (mL/Min)	0.24	0.24	0.25	0.25
Source T-WAVE Parameters	Automated	Automated	Automated	Automated
Collision Cell T-WAVE Parameters	Automated	Automated	Automated	Automated

The LC-column eluent was directed to the ESI source of a Quattro Premier triple quadrupole mass spectrometer (Waters, Manchester, UK) operated in positive ion mode, with nitrogen as the nebulizing and desolvation gas, and argon as the collision gas. The system was calibrated for specific acquisition modes including MS+, precursor ion scanning (PIS) at m/z 184 and 369, and multiple

reaction monitoring (MRM) for targeted analyses. Capillary voltages, collision energies and gas flows were optimized for each acquisition mode (see table "Instrument parameters"). TGs were assayed using direct MS+ scanning, while PIS modes were employed for selective detection of PC, LPC, and SM (PIS184), as well as CE (PIS369). Cer was detected with a targeted analysis using selected reaction monitoring (SRM). For the SRM-transitions, the proton adducts [M + H+] of the different Cer species served as precursor ions and the product ions were m/z 264 for doubly dehydrated sphingosine (SPB 18:1,O2).

Lipid identification and quantification

Raw data were processed using QuanLynx V 4.1 software (Waters Lab Informatics, UK). Lipid species were identified based on their mass-to-charge (*m/z*) ratios, relative retention times, and MS/MS fragmentation patterns, with specific attention paid to the proton [+H+] adducts of ceramide and phospholipid species, and ammonium [+NH4+] adducts of TG and SE species. Custom quantification methods in QuanLynx were developed for each lipid class, where individual lipid species were identified based on peak *m/z* (tolerance ± 0.5 Da) and retention time (tolerance ± 0.2 min) relative to spiked-in internal lipid standards.

Using the chromatographic system employed, various lipid species gave rise to multiple signals with equal *m/z* but different retention times. After carefully considering potential isotopic overlaps (M+1, M+2) from other lipid signals, peaks that were deemed monoisotopic (M+0) signals, and were consistently time-resolved, were assigned as isobaric lipid species (e.g. PC 38:5a and PC 38:5b). Isobaric signals likely arise from species with identical total carbon and double bond numbers, but different fatty acyl constituents (e.g. PC 18:0_20:5 or PC 18:1_20:4 for PC 38:5), which may vary in their chromatographic retention.

The peak areas of identified lipid species were then integrated. The integration parameters in QuanLynx (smoothing, apex track, and window extent) were optimised per analyte to minimise the effects of analyst subjectivity during data processing. Nevertheless, manual corrections in peak picking were applied when necessary. Only the monoisotopic (M+0) signals were considered.

The integrated peak areas and peak metadata were exported to Microsoft® Excel v. 16.87 (Microsoft Corporation, Richmond, WA, USA), the data was organized and exported as a .csv-file. The datasets were then processed using an Orange3, v. 3.34.1 (University of Ljubljana, Slovenia) pipeline to carry out blank corrections, accounting for background contaminants, and to calculate lipid abundances based on the peak areas of spiked-in internal lipid standards. These abundances were then normalized to the protein content of the original sample.

A full list of detected lipid species, their retention times, and concentrations in lipoproteins is available in *Supplemental Table 6*.

Gas chromatography analyses of plasma total fatty acids

Fatty acid composition of plasma total lipids was determined by gas chromatography. Internal standard fatty acid 13:0 (20 µg = 93 nmol) was mixed with a 50 µl aliquot of plasma. The samples were evaporated near to dryness under a nitrogen stream just prior to transmethylation,

performed according to Christie (3). Briefly, the samples were reconstituted in 2 mL 1% methanolic H_2SO_4 (Sigma-Aldrich, St. Louis, MO, USA), with 1 mL hexane (Merck, VWR Finland) as a cosolvent. The sample vials were flushed with nitrogen gas and sealed, after which they were heated for 110 min at 96 °C. After adding 1.5 mL millipore water, the resulting fatty acid methyl esters (FAME) and dimethylacetals (DMA) were extracted twice with 4 mL hexane and the combined extracts were dried over anhydrous Na_2SO_4 (Merck, VWR Finland). The dried extracts were concentrated by evaporation under a nitrogen stream and stored at -80 °C for a maximum of 3 weeks until analysed.

The FAME-extracts were analysed by two parallel GC-pipelines for identification and quantitation. A GCMS-QP2010 Ultra (Shimadzu Scientific Instruments, Kyoto, Japan) with electron impact (EI) mass detector (MSD) was employed for structure identification, and Shimadzu GC-2010 Plus gas chromatograph (Shimadzu Scientific Instruments) with flame ionization detector (FID) for quantification. Both systems employed a Zebron ZB-wax capillary column (30 m, ID 0.25 mm, film thickness 0.25 µm, Phenomenex, Torrence, CA, USA). A volume of 4 µL was injected with a split ratio of 1:25. The injectors were set at 250°C, and the FID and MSD interphases were set at 250°C and 200°C, respectively. Helium was used as the carrier gas (1.8 mL/min for the FID and 1.0 mL/min for the MSD equipment). The initial oven temperature of 180°C was held for 8 minutes, programmed to rise at 3°C/min to a final temperature of 210°C, which was held for 40 minutes.

For FAME-identification, the resulting GC-MS traces were processed and extracted using GCMSsolution V4.30 software (Shimadzu Scientific Instruments). The FAMEs were identified based on retention time, m/z, and EIMS-fragmentation comparisons with authentic standards, as well as published reference spectra (hyperlink).

For quantification of the identified FAMEs and DMAs, FID traces were processed and integrated using GCsolution V2.42.00 software (Shimadzu Scientific Instruments). FID responses were corrected according to theoretical response factors and FAMEs were then quantified based on calibrations with quantitative authentic standards. Total fatty acid concentrations in plasma were calculated based on the spiked-in internal standard fatty acid 13:0. A full list of detected fatty acids, plasma concentrations and relative change are available in *Supplemental Table 2*.

Procedure for estimating the number of EPA-containing lipid molecules in lipoprotein particles

The following steps outline the process used to estimate the number of lipid molecules containing or not containing EPA in each lipoprotein class (VLDL, LDL, HDL). The process involves combining data from NMR and LC-MS analyses, as outlined below.

Determine the number of circulating lipoprotein particles.

Plasma concentrations of circulating lipoprotein classes (VLDL, LDL, HDL) were quantified using NMR spectroscopy (see *Supplemental Table 4*). The total number of lipoprotein particles for each class within the plasma volume was obtained by multiplying the lipoprotein concentration by Avogadro's number ($N_A = 6.022 \times 10^{23}$ particles/mol):

$$\text{Lipoprotein particles} \;\; \mathrm{L}^{-1} = [\text{Lipoprotein}] \times N_A$$

Example with LDL:

$$[\mathrm{LDL}] = 0.00108\,\mathrm{mM} = 1.08 \times 10^{-6}\,\mathrm{mol/L}$$

$$\mathrm{LDL\ particles} \times \mathrm{L}^{-1} = 1.08 \times 10^{-6}\,\mathrm{mol/L} \times 6.022 \times 10^{23}\,\mathrm{particles/mol} = 6.502 \times 10^{17}\,\mathrm{particles/L}$$

Determine the number of circulating lipid molecules within each lipoprotein class.

The concentrations of specific lipid classes - CE, PL and TG - associated with each lipoprotein class were measured using NMR spectroscopy, as presented in *Supplemental Table 4*. For each lipid class within each lipoprotein class, the number of lipid molecules per litre of plasma was determined by multiplying the lipid concentration by Avogadro's number (NA = 6.022 × 10^{23} molecules/mol):

$$\text{Lipid molecules} \;\; \mathrm{L}^{-1} = [\text{Lipid}] \times N_A$$

Example with LDL-TG:

$$[\mathrm{LDL} - \mathrm{TG}] = 0.128\,\mathrm{mM} = 1.28 \times 10^{-4}\,\mathrm{mol/L}$$

$$\mathrm{LDL} - \mathrm{TG\ molecules} \times \mathrm{L}^{-1} = 1.28 \times 10^{-4}\,\mathrm{mol/L} \times 6.022 \times 10^{23}\,\mathrm{molecules/mol} = 7.707 \times 10^{19}\,\mathrm{molecules/L}$$

Calculate the number of lipid molecules per lipoprotein particle

The average number of lipid molecules per lipoprotein particle was calculated for each combination of lipid class (PL, CE, TG) and lipoprotein class (VLDL, LDL, HDL). This was achieved by dividing the total number of lipoprotein-associated lipid molecules per litre by the corresponding number of lipoprotein particles per litre:

$$\text{Lipid molecules per particle} = \frac{\text{Lipid molecules} \;\; \mathrm{L}^{-1}}{\text{Lipoprotein particles} \;\; \mathrm{L}^{-1}}$$

Example with LDL-TG:

$$\mathrm{LDL\ particles} \times \mathrm{L}^{-1} = 6.502 \times 10^{17}\,\mathrm{particles/L}$$

$$\mathrm{LDL} - \mathrm{TG\ molecules} \times \mathrm{L}^{-1} = 7.707 \times 10^{19}\,\mathrm{molecules/L}$$

$$\mathrm{LDL} - \mathrm{TG\ molecules\ per\ particle} = \frac{7.707 \times 10^{19}\,\mathrm{molecules/L}}{6.502 \times 10^{17}\,\mathrm{particles/L}} \approx 118.5\,\mathrm{TG\ molecules/particle}$$

Determine the fraction of EPA-containing lipid species in each lipid class

Isolated fractions of VLDL, LDL, and HDL were subjected to LC-MS analysis to identify and quantify individual lipid species within each lipoprotein class. To estimate the proportion of EPA-containing lipid molecules within each lipid class, two criteria were applied:

1. Theoretical capacity for EPA incorporation: only lipid species with a total acyl carbon number is ≥20 and a minimum of 5 double bonds were considered.
2. Post-supplementation increases: Lipid species were classified as EPA-containing if their abundance increased by more than 30% following icosapent ethyl (IPE) supplementation, comparing measurements from Day 0 to Day 28.

For each lipid class within VLDL, LDL and HDL, the sum of abundances of lipid species meeting both criteria was calculated. This sum was then divided by the total abundance of all lipid species within the respective lipid class to determine the fraction of EPA-containing lipids.

$$\text{Fraction of EPA} - \text{containing lipids} = \frac{\sum(\text{EPA} - \text{containing lipid species})}{\sum(\text{all lipid species})}$$

Notably, for PL calculations SM was included in the total lipid sum despite not containing EPA to facilitate conversion of the NMR data, which also accounted SM.

Example with LDL-TG:

$$\text{EPA} - \text{containing TG} = 29.7\,\text{pmol/ug protein}$$

$$\text{Non} - \text{EPA} - \text{containing TG} = 261.1\,\text{pmol/ug protein}$$

$$\text{Total TG} = 29.7 + 261.1 = 290.8\,\text{pmol/ug protein}$$

$$\text{Fraction of EPA} - \text{containing TG} = \frac{29.7}{290.8} \approx 0.1021\,(10.21)$$

Estimate the number of EPA-containing lipid molecules per lipoprotein particle.

Finally, the number of EPA-containing lipid molecules per lipoprotein particle was estimated for each combination of lipid class and lipoprotein class. This was accomplished by multiplying the fraction of EPA-containing lipid molecules in a lipid class (determined in Step d) by the number of each type of lipid molecules per particle (calculated in Step c):

$$\text{Number of EPA} - \text{containing lipid molecules per particle} =$$

$$\text{Fraction of EPA} - \text{containing lipid} \times \text{Lipid molecules per particle}$$

Example with LDL-TG:

$$\text{Fraction of EPA} - \text{containing lipids for LDL} = 0.1021\,(10.21)$$

$$\text{LDL} - \text{TG molecules per particle} \approx 118.5\,\text{TG molecules/particle}$$

$$\text{EPA} - \text{containing TG per LDL particle} = 0.1021 \times 118.5 \approx 12.09\,\text{TG molecules/particle}$$

Summary of iterative calculations

This procedure systematically performed calculations for each lipid class (PL, CE, TG) within each lipoprotein class (VLDL, LDL, HDL). For every combination, plasma concentrations were quantified, converted to absolute numbers using Avogadro's number, and normalized per lipoprotein particle. EPA incorporation was assessed based on theoretical capacity and supplementation-induced abundance changes, resulting in the estimation of EPA-containing lipid molecules per particle.

Statistical methods

Normality tests

The normal distribution of variables was tested using the D'Agostino-Pearson omnibus K2 test using GraphPad Prism v10.1.2 (LA Jolla, CA, USA). This test evaluates the skewness and kurtosis of the data to determine if it deviates significantly from a normal distribution.

Group differences

Statistical significances of differences between groups (time points) were assessed using paired multiple *t*-tests (LIMMA) on $\log_2$ transformed data. Multiple hypothesis correction was applied using the false discovery rate (FDR). Analyses were performed using the PolySTest tool (4). Before the group mean comparisons, outliers were removed following an outlier analysis performed with ROUT-method (Q=0.01) using GraphPad Prism v10.1.2 (La Jolla, CA, USA). All data were combined into a single .csv-file, and group differences for all individual variables were calculated in bulk.

For certain pairwise comparisons, a paired, two-tailed Student's *t*-test was performed using GraphPad Prism (v10.1.2, La Jolla, CA, USA).

Analysis of variance

In specific cases, One-way ANOVA was used to compare group means. For *post hoc* analyses a Two-stage linear step-up method of Benjamini, Krieger and Yekutieli for multiple comparisons test (FDR) was implemented to identify specific group differences when significant main effects were found. Analyses were carried out using GraphPad Prism v10.1.2 (LA Jolla, CA, USA).

Correlation Analyses

Spearman's correlations were used to assess the relationships between variables. For datasets with 17 or fewer pairs of values, exact *p*-values were computed by considering all possible permutations of the data. For larger datasets, approximate *p*-values were computed by deriving a *t* ratio from the Spearman rank correlation coefficient (Rs) and calculating the *p*-value from this t ratio. This method handles ties and ensures accurate results for larger datasets. The analyses were implemented using GraphPad Prism v10.1.2 (LA Jolla, CA, USA).

Time series clustering of lipoprotein lipid species

Time series clustering analyses were performed using VSClust (5), available online at VSClust Bitbucket Repository. VSClust employs an improved fuzzy c means clustering method that avoids arbitrary averaging and assigns individual fuzzifier scores to each feature at all time points based on the feature centroid. The analyses were implemented in RStudio (Version 2023.12.1, build 402, Posit Software, PBC) employing R Version 4.3.2 (The R Foundation for Statistical Computing).

As data input, a comprehensive table of lipoprotein lipid species abundances (pmol/µg) across all time points in lipoprotein classes was compiled. Variables with more than 50% missing data at any time point were excluded. Remaining missing values were imputed by time point using the k-nearest neighbors (kNN) method with the *VIM* package. The data was then $\log_2$-transformed to stabilize variance and reduce skewness and prepare it for clustering. The pre-processed and log-normalized data was reshaped for clustering analysis by the *vsclust* package.

Parameters for clustering analysis were set, including the number of replicates per condition, the number of conditions, and whether the data were paired. The *PrepareForVSClust* function was

used to run statistical analysis and estimate individual variances. The optimal number of clusters was estimated using *estimClustNum*, and clustering was performed using both *VSClust* and standard fuzzy c-means methods. Clustering results were analysed to identify significant clusters based on membership values. Mean centroid lines for each cluster were calculated and visualized using ggplot2, showing the temporal patterns of lipidomic profiles across the different time points.

Principal component analyses of lipoprotein lipidomes

Principal component analyses were implemented in RStudio (Version 2023.12.1, build 402, Posit Software, PBC) employing R Version 4.3.2 (The R Foundation for Statistical Computing).

Variables with more than 50% missing data at any time point were excluded. Remaining missing values were imputed by time point using the k-nearest neighbors (kNN) method (k=5) with the *VIM* package. The data was then $\log_2$-transformed to stabilize variance and reduce skewness.

PCA was then performed on the $\log_2$-normalised lipid abundances (pmol/ug protein) using the *prcomp* function of the *stats* package, with centering and scaling of the data to ensure comparability across variables.

Linear mixed modelling of lipoprotein lipidome principal components

Linear mixed models (6) were employed to assess the contributions of fixed and random effects on the variance of lipoprotein lipidome principal components. The analyses were implemented in RStudio (Version 2023.12.1, build 402, Posit Software, PBC) employing R Version 4.3.2 (The R Foundation for Statistical Computing).

For each of the 10 first principal components, a mixed effects model was fitted using the *lmer* function from the *lme4* package (7). The model formula included Time (i.e., the four sampling time points, reflecting IPE-induced effects) as a fixed effect, and Subject (i.e., the individual participants) as a random effect, which accounted for repeated measures (four observations per subject):

$$PC \sim Time + (1|Subject)$$

This structure enabled the examination of the supplement effect while accounting for variability among subjects.

The model was fitted using Restricted Maximum Likelihood (REML) estimation, with a maximum of 100 iterations and convergence criteria of a parameter estimate change below 1e-6. Gradient and Hessian checks were performed to verify convergence, based on the first and second derivatives of the log-likelihood function, respectively.

The fitted mixed models returned the estimated effects of treatment on each principal component, including fixed effects coefficients and random effects variance components. These estimates were then used to assess the contribution of fixed and random effects on the total variance in the principal components. Marginal and conditional R-squared values were computed using the *r.squaredGLMM* function from the *MuMIn* package, to assess the variance explained by fixed and random effects, respectively (1). Marginal R-squared reflects the variance explained by the fixed effects alone, while conditional R-squared indicates the variance explained by both fixed and random effects. These proportions, alongside the absolute variances derived from the PCA, were used to calculate the explained variance attributable to the fixed and random effects of the total variance.

Hierarchical clustering of lipoprotein lipidome principal components

Hierarchical clustering was applied to explore patterns and structure within the log_2-normalized lipid species abundances across VLDL, LDL, and HDL lipidomes, as well as their combined dataset. This unsupervised approach evaluates relationships and groupings based on the overall similarity between samples, without making predefined assumptions about the data structure. The analyses were conducted in RStudio (Version 2023.12.1, build 402, Posit Software, PBC), employing R Version 4.3.2 (The R Foundation for Statistical Computing).

PCA was conducted on each log_2-transformed, centered, and scaled dataset using the *prcomp* function from the *stats* package to reduce dimensionality, limiting the analysis to the first 15 principal components (PCs) to achieve >80% cumulative explained variance. This step preserves the primary variance while minimizing noise from lesser components. A Euclidean distance matrix was generated from the PCA scores using the *dist* function from the *stats* package, and hierarchical clustering was applied to this distance matrix using the *hclust* function with the 'ward.D2' method.

Ward's method (Ward.D2) was employed as the linkage criterion for hierarchical clustering, minimizing the variance within clusters as observations are grouped together. The resulting dendrograms were plotted with sample identifiers as labels. The height of each branch in the dendrogram represents the distance between clusters, indicating the similarity between samples' lipidomic profiles at different time points. The branching process illustrates how samples are successively merged into clusters based on their similarities, creating a hierarchical structure that reveals the relationships among the samples. Similar clustering results were obtained when applying alternative linkage methods, including complete and average linkage.

Cophenetic correlation coefficients were calculated for each dataset to assess the fit of the hierarchical clustering to the original distance matrix. The cophenetic correlation quantifies how faithfully the dendrogram represents the pairwise distances between observations from the original dataset. Calculations were performed using the *cophenetic()* function of the *stats* package, which computes the cophenetic distances from the dendrogram, and these were then correlated with the original Euclidean distances using Pearson's correlation.

Distance calculations of lipoprotein lipidome principal components

Euclidean distances were calculated based on the principal component scores from the first 15 principal components of PCA analysis, which were performed on the log_2-normalized lipid species abundances for HDL, LDL, and VLDL lipidomes, as well as an aggregate dataset combining all lipid species for collective analysis. The first 15 components were selected to capture >80% of the explained variance in each dataset. Distance matrices of the PCA scores were generated using the *dist* function from the *stats* package in R.

To assess the contribution of IPE-supplementation (Time) and intersubject variability to the overall variance of lipoprotein lipidomes, pairwise comparisons of the distances between observations (samples) were conducted, comparing relationships across four groupings:

Mean distances: distances between all observations in each dataset were calculated and averaged.

Within Subject: distances between observations from the same individual were calculated to assess the consistency of lipid profiles across time points for each subject.

Between Subjects: distances between observations from one subject and those from other subjects were calculated to assess interindividual variability.

Within Time Point: distances among samples taken at the same time point were calculated separately for each time point, and then aggregated, to evaluate variance associated with IPE-supplementation at a group level

These distances were summarized and compared using boxplots, and statistical significance between groups was evaluated using pairwise Wilcoxon rank-sum test to compare distributions across the different distance groups.

Sparse partial least squares-discriminant analyses of lipoprotein lipidomes

Sparse partial least squares-discriminant analysis (sPLS-DA) is a supervised statistical method to identify variables that can best discriminate between different groups or classes in the dataset (8). The algorithm uses known class labels (e.g. time points, lipoproteins) to supervise the learning process and maximize the separation between the predefined groups.

sPLS-DA of lipoprotein lipidomes were conducted using the Statistical Analysis [one factor] module of Metaboanalyst 6.0 (9). The input was a .csv-file containing lipid metabolite abundances (pmol/µg), with samples grouped by lipoprotein (HDL, LDL and VLDL) and time point (Day 0, 7, 28, and 35). Variables with more than 50% missing values were filtered out and the remaining missing values were imputed using the k-nearest neighbors (kNN) method. Data was further filtered using an interquartile range (IQR) -based variance filter, which removed 10% of variables with near-constant variance. Prior to analysis, the data were $\log_{10}$-transformed and scaled.

For the sPLS-DA analysis, the number of latent vectors (components) was limited to 3 and the number of variables with highest variance was set to 15 per component. The sPLS-DA was then carried out using standard Metaboanalyst 6.0 settings. The performance of the sPLS-DA model was evaluated using cross validations (CV). The resulting scores were exported as a JSON file, imported to RStudio (V.2023.12.1, build 402, Posit Software, PBC) employing R Version 4.3.2 (The R Foundation for Statistical Computing) using the *jsonlite* package, and a 3D plot was generated using the *plotly* and *shiny* packages.

Uniform manifold approximation projection analysis of lipoprotein lipidomes

Uniform Manifold Approximation and Projection (UMAP) is a dimensionality reduction technique that has gained popularity for its ability to visualize high-dimensional data in a lower-dimensional space, often used for data exploration and visualization. The primary goal of UMAP is to preserve the global structure of the data while also maintaining local relationships (10). It is particularly useful for visualizing complex datasets in two or three dimensions, making patterns and clusters more apparent. Compared to Principal Component Analysis (PCA), a linear dimensionality reduction technique, UMAP can capture more complex, non-linear relationships.

UMAP analyses were implemented in RStudio (V.2023.12.1, build 402, Posit Software, PBC) employing R Version 4.3.2 (The R Foundation for Statistical Computing).

As data input, a comprehensive table of the abundances of VLDL, LDL and DL lipid species (pmol/ug protein). Variables with more than 50% missing data at any time point were excluded. Remaining missing values were imputed by time point using the k-nearest neighbors (kNN) method with the *VIM* package. The data was then $\log_{2}$-transformed to stabilize variance and

reduce skewness, followed by z-scaling. The pre-processed data was then analysed using the *umap* package.

UMAP was performed using the umap function with the following parameters: a seed was set for reproducibility; *n_neighbors* = 15 (Number of neighboring points used in local approximations of manifold structure); *min_dist* = 0.1 (Minimum distance between points in the low-dimensional representation); *metric* = "euclidean" (Distance metric used to compute the distances in the input space); *n_components* = 2 (Number of dimensions for the UMAP projection). The resulting projection was visualized using ggplot2 and further processed in Incscape (version 1.3.2; Incscape project).

Machine learning methods to predict lipoprotein binding to aortic proteoglycans and LDL aggregation

We employed machine learning analyses to generate regression models, which predict lipoprotein binding to aortic proteoglycans or LDL aggregation propensity based on plasma and lipoprotein metabolites. Tree-based machine learning models offer significant advantages over univariate tests (e.g., *t*-tests, linear regressions) by allowing the inclusion of a large number of variables in one model, thereby improving power in small sample sizes and providing robustness to non-normal distributions of variables (11, 12). The XGBoost algorithm, which employs extreme gradient boosting (12), is widely used for its accuracy and efficiency in various omics analyses (11).

XGBoost machine learning analyses were implemented in RStudio (V.2023.12.1, build 402, Posit Software, PBC) employing R Version 4.3.2 (The R Foundation for Statistical Computing).

As data input, a comprehensive table of the abundances of HDL, LDL, and VLDL lipid species (pmol/µg protein), plasma biomarkers (mM) across all time points, and baseline clinical parameters were compiled. Variables with more than 50% missing data at any time point were excluded. Remaining missing values were imputed by time point using the k-nearest neighbors (kNN) method with the VIM package. The data was then $\log_2$-transformed to stabilize variance and reduce skewness. Models for predicting lipoprotein binding to proteoglycans or LDL aggregation were then generated. Separate models were constructed using the full comprehensive dataset and using only the LDL-lipidome and clinical data.

Features and labels were prepared separately for both training and testing datasets. Features included the abundances of lipid species, plasma biomarkers, and clinical parameters, while labels were the target outcomes such as lipoprotein binding to proteoglycans or LDL aggregation propensity. DMatrix objects, which are a highly optimized data structure provided by the *xgboost* package for training and prediction, were created for both training and testing sets to optimize performance with XGBoost.

We employed a nested cross-validation framework (12). Our analysis involved randomly splitting the dataset into 30% test and 70% training sets using the caret package's *createDataPartition* function. Each training set was further subjected to a 5-fold cross-validation process for hyperparameter tuning. Two random variables were introduced into the dataset as benchmarks to ensure the robustness of our feature selection.

The primary evaluation metrics for the regression models included Root Mean Squared Error (RMSE), Mean Absolute Error (MAE), and Explained Variance. These metrics were calculated on the

test set to assess model performance. The nested cross-validation was iterated 200 times to ensure stability and reliability of the results. In each iteration, feature importance was ranked and averaged across all iterations to identify the most significant predictors. The results from the test sets across iterations were averaged to provide comprehensive performance metrics. To prevent overfitting, we conducted permutation tests where the target variable was randomly shuffled, and the models were re-evaluated using the same nested cross-validation procedure. This approach ensured that our models were genuinely predictive and not merely fitting noise in the data.

The mean RMSE, MAE, and Explained Variance were calculated across all iterations and indicated that the nested cross-validation process provided robust performance. The feature importance analysis identified key metabolites significantly contributing to the predictive model, averaged over 200 iterations, ensuring consistency and reliability of the identified predictors. Feature importance was assessed using the gain metric, which was plotted to visually represent the contribution of each feature to the model. This visualization helps to understand which features have the most significant impact on the model's predictions, highlighting the variables that are most influential in predicting lipoprotein binding to aortic proteoglycans or LDL aggregation propensity.'

SUPPLEMENTAL REFERENCES

1. Nakagawa S, and Schielzeth H. A general and simple method for obtaining R2 from generalized linear mixed-effects models. *Methods in Ecology and Evolution.* 2013;4(2):133-42.

2. Liebisch G, Fahy E, Aoki J, Dennis EA, Durand T, Ejsing CS, et al. Update on LIPID MAPS classification, nomenclature, and shorthand notation for MS-derived lipid structures. *Journal of Lipid Research.* 2020;61(12):1539-55.

3. Christie WW. *Preparation of ester derivatives of fatty acids for chromatographic analysis.* Invergowrie, Dundee, Scotland: Oily Press, Dundee; 1993.

4. Schwämmle V, Hagensen CE, Rogowska-Wrzesinska A, and Jensen ON. PolySTest: Robust Statistical Testing of Proteomics Data with Missing Values Improves Detection of Biologically Relevant Features. *Molecular & Cellular Proteomics.* 2020;19(8):1396-408.

5. Schwämmle V, and Jensen ON. VSClust: feature-based variance-sensitive clustering of omics data. *Bioinformatics.* 2018;34(17):2965-72.

6. Bates D. Computational methods for mixed models. *Vignette for lme4.* 2011;1045:1046.

7. Bates D, Mächler M, Bolker B, and Walker S. Fitting Linear Mixed-Effects Models Using lme4. *Journal of Statistical Software.* 2015;67(1):1 - 48.

8. Lê Cao K-A, Boitard S, and Besse P. Sparse PLS discriminant analysis: biologically relevant feature selection and graphical displays for multiclass problems. *BMC Bioinformatics.* 2011;12(1):253.

9. Pang Z, Lu Y, Zhou G, Hui F, Xu L, Viau C, et al. MetaboAnalyst 6.0: towards a unified platform for metabolomics data processing, analysis and interpretation. *Nucleic Acids Research.* 2024;52(W1):W398-W406.

10. McInnes L, Healy J, and Melville J. Umap: Uniform manifold approximation and projection for dimension reduction. *arXiv preprint arXiv:180203426.* 2018.

11. Liebal UW, Phan ANT, Sudhakar M, Raman K, and Blank LM. Machine Learning Applications for Mass Spectrometry-Based Metabolomics. *Metabolites.* 2020;10(6):243.

12. Chen T, and Guestrin C. *Proceedings of the 22nd ACM SIGKDD International Conference on Knowledge Discovery and Data Mining*. ACM; 2016.
